# Effectiveness and safety of acupuncture and related therapies for pediatric asthma: a systematic review and meta-analysis

**DOI:** 10.3389/fmed.2025.1626830

**Published:** 2025-07-16

**Authors:** Jixuan Wang, Shun Fan, Kaihan Su, Jiacheng Zhang, Xuanjun Lu, Shengxuan Guo, Siyuan Hu

**Affiliations:** ^1^Department of Clinical Trial Center, First Teaching Hospital of Tianjin University of Traditional Chinese Medicine, Tianjin, China; ^2^National Clinical Research Center for Chinese Medicine Acupuncture and Moxibustion, Tianjin, China; ^3^Department of Tuina, First Teaching Hospital of Tianjin University of Traditional Chinese Medicine, Tianjin, China

**Keywords:** acupuncture, acupuncture-related therapies, pediatric asthma, meta-analysis, systematic review

## Abstract

**Objectives:**

This study aims to investigate the efficacy and safety of acupuncture and related therapies as an adjunct to standard treatment (ST) in children with asthma.

**Methods:**

Randomized controlled trials (RCTs) comparing acupuncture combined with ST versus ST alone for pediatric asthma have been included. 8 databases and 3 clinical trial registries were searched, with the search completed up to January 31, 2025. The risk of bias in the included studies was assessed using the Risk of Bias 2 (RoB 2) tool. Data from the included studies were analyzed using R software version 4.4.2. The quality of evidence was evaluated using the Grades of Recommendations, Assessment, Development, and Evaluation (GRADE) approach.

**Results:**

A total of 16 randomized controlled trials involving 1,675 participants have been included. Compared to ST, the addition of acupuncture has significantly improved the percent predicted values of forced expiratory volume in 1 second (FEV1pred%) [MD = 6.02, 95% CI (1.28, 10.76), *p* = 0.0128], No significant effect on forced expiratory volume in 1 second/forced vital capacity (FEV1/FVC) has been observed [MD = 3.36, 95% CI (−0.76, 7.48), *p* = 0.1097]. In addition, acupuncture has significantly reduced serum immunoglobulin E (IgE) levels [SMD = −0.88, 95% CI (−1.21, −0.55), *p* < 0.0001]. It has significantly increased serum immunoglobulin A (IgA) levels [MD = 0.31, 95% CI (0.22, 0.41), *p* < 0.0001]. It has also significantly improved serum immunoglobulin G (IgG) levels [MD = 1.71, 95% CI (1.39, 2.02), *p* < 0.0001]. Acupuncture has significantly increased peak expiratory flow (PEF) [MD = 3.15, 95% CI (1.16, 5.14), *p* = 0.0019]. It has significantly reduced serum interleukin-4 (IL-4) levels [SMD = −2.40, 95% CI (−2.75, −2.05), *p* < 0.0001]. Acupuncture has significantly decreased eosinophil (EOS) levels [MD = −1.06, 95% CI (−1.68, −0.43), *p* = 0.0010]. However, acupuncture has shown no significant effect on the pediatric asthma quality of life questionnaire (PAQLQ) scores [MD = 0.01, 95% CI (−0.39, 0.40), *p* = 0.9778].

**Conclusion:**

Acupuncture has shown positive effects on certain serum immune and inflammatory biomarkers and FEV1 in pediatric asthma. It has not shown beneficial effects on FEV1/FVC. A substantial proportion of the evidence has been of low quality, and confidence in the results has been downgraded due to a serious risk of bias and inconsistency. The actual effects may differ substantially from the findings of this study. High-quality randomized controlled trials are still needed to confirm these findings in the future.

**Systematic review registration:**

https://www.crd.york.ac.uk/PROSPERO/view/CRD420251039313.

## 1 Introduction

Asthma is a major non-communicable disease that affects people of all ages. Asthma is characterized by chronic airway inflammation that leads to bronchoconstriction, edema, increased mucus production, and airway hyperreactivity. Clinical symptoms include wheezing, shortness of breath, chest tightness, and cough. A study published in *The Lancet* reviewing the disease burden from 1990 to 2019 reported that asthma affected approximately 262 million people and caused 455,000 deaths (1). With the growth of the global population, the number of people with asthma is projected to reach 400 million by 2025 (2).

According to the World Health Organization, asthma is the most common chronic disease in children and ranks among the top 20 global causes of disability-adjusted life years in children (3). Although asthma-related mortality is declining, the prevalence of pediatric asthma continues to rise globally, reaching approximately 81 million children by 2019 (4). In the United States, 8.3% of children have asthma (5), while the prevalence among children living in urban areas of China is 3.02% (6). The risk of pediatric asthma is associated with multiple factors, including genetics, environment, perinatal conditions, and drug exposure (7–10). Unlike in adults, the clinical manifestations of asthma vary by age in children (11). Therefore, the management of children with asthma is adjusted according to their age group. The 2024 Global Initiative for Asthma (GINA) Guidelines recommend low-dose inhaled corticosteroids (ICS) as the preferred initial treatment for children aged 6–11, in combination with Short-Acting β2-Agonist (SABA) as needed. For adolescents, GINA recommends the use of low-dose ICS combined with formoterol (12). However, asthma medications in children may cause side effects, such as reduced growth velocity and adrenal insufficiency associated with long-term ICS use (13).

Acupuncture is a therapy originating from traditional Chinese medicine. Traditional acupuncture, also known as manual acupuncture, involves the insertion of needles into specific acupoints by hand. With the evolution of acupuncture tools, several non-traditional techniques have emerged, including electroacupuncture (EA), acupoint catgut embedding (ACE), transcutaneous electrical acupoint stimulation (TEAS), laser acupuncture (LA), and press needle (PN), which stimulate acupoints in specialized ways. A network meta-analysis showed that acupoint catgut embedding was the most effective in improving patients’ asthma control test (ACT) scores (14). Acupuncture is increasingly being adopted in clinical practice for pediatric asthma management in many countries. The potential mechanisms by which acupuncture alleviates asthma are multifaceted, including balancing T helper type 1 cells (Th1)/T helper type 2 cells (Th2) and Regulatory T cells (Treg)/T helper type 17 cells (Th17) immune responses to suppress airway inflammation and downregulating autophagy-related proteins in lung tissue to reduce symptoms and airway remodeling (15). A Cochrane systematic review (16) concluded that there is insufficient evidence to recommend acupuncture for pediatric asthma. However, Liu et al. (17) found that acupuncture may improve peak expiratory flow (PEF) and weekly variability of PEF in children with asthma, although its effects on other outcomes remain unclear. Zhang et al. (18) found no sufficiently convincing evidence to support the effectiveness of LA in treating pediatric asthma. This research conducts a comprehensive assessment of the effectiveness and safety profile of acupuncture in the management of pediatric asthma, drawing evidence exclusively from randomized controlled trials (RCTs).

## 2 Methods

### 2.1 Registration and protocol

This study has been registered in the PROSPERO database (CRD420251039313) and was reported in strict accordance with the Preferred Reporting Items for Systematic Review and Meta-Analysis (PRISMA) 2020 guidelines (19).

### 2.2 Study search

This study collected published research by searching 4 international databases (PubMed, EMBASE, Cochrane Library, and Web of Science) and 4 Chinese databases (Chinese National Knowledge Infrastructure, Chinese Biological Medicine, WanFang, and VIP database). We supplemented the search by exploring ClinicalTrials.gov, the Chinese Clinical Trial Registry, the International Traditional Medicine Clinical Trial Registry, and OpenGrey to collect unpublished negative results or ongoing studies. The search cutoff date was set to January 31, 2025, with no filters applied and no language restrictions on the study. A combination of subject headings and free text search terms was used, with the search strategy for each database detailed in Supplementary File 1. The detailed inclusion and exclusion criteria for the study are provided below, along with justifications.

#### 2.2.1 Types of participants

We included children with asthma under the age of 18, with no restrictions on the clinical phenotype of asthma. Those diagnosed with classical asthma (CA), cough variant asthma (CVA), chest tightness variant asthma, and exercise-induced asthma were all included. The 2024 GINA Guidelines also began to focus on the management of the CVA clinical phenotype. Although CVA does not exhibit clear symptoms or signs such as wheezing or shortness of breath, its pathogenesis is highly similar to that of CA, with both presenting airway hyperresponsiveness. CVA is only less severe in degree, and both involve Th2-associated cytokines and eosinophilic inflammation (20). We excluded children with comorbid conditions (such as allergic rhinitis, mycoplasma infection, allergic purpura, etc.) to ensure the consistency of clinical features among the asthma patients.

#### 2.2.2 Types of interventions

The interventions included both traditional and non-traditional acupuncture, such as MA, EA, ACE, TEAS, LA, and PN. The experimental group received acupuncture combined with standard treatment (ST), which included both pharmacological and non-pharmacological therapies, such as ICS and SABA. The acupuncture intervention period was greater than 1 week to exclude patients with acute asthma.

#### 2.2.3 Types of control

The control group received either a wait-list treatment, no treatment, or placebo stimulation. Although this included studies with lower methodological quality, some non-traditional acupuncture methods could not be blinded. Therefore, we decided that the control group could either not receive treatment or be placed on a waiting list for treatment. The control group received the same ST as the experimental group.

#### 2.2.4 Types of outcomes

The primary outcome measure was the percent predicted value of forced expiratory volume in 1 second (FEV1pred%), and the secondary outcomes included forced expiratory volume in 1 second/forced vital capacity (FEV1/FVC), serum immunoglobulin E (IgE) levels, serum immunoglobulin A (IgA) levels, serum immunoglobulin G (IgG) levels, PEF, serum interleukin-4 (IL-4) levels, peripheral blood eosinophil (EOS) counts, and the pediatric asthma quality of life questionnaire (PAQLQ). There was no time limit for measuring any of the outcomes. We did not pool the efficacy rates, as most efficacy rates were based on the conversion of Traditional Chinese Medicine symptom scores into ordinal data, calculating the proportion of effective cases out of the total. This may have resulted in a loss of statistical power for the original data. If no blinding was used in the study, the credibility of this outcome was lower.

#### 2.2.5 Types of studies

Only RCTs were included, as RCTs are the gold standard for explaining causal relationships. In terms of study design, only parallel and load designs were included, and crossover designs were excluded due to the uncertainty of the washout period before acupuncture treatment.

### 2.3 Study selection

We used EndNote X20 to manage all the retrieved studies and removed duplicates using EndNote. Two researchers (JXW and JCZ) independently screened the study, and the results from each stage were compared. If discrepancies arose, a third reviewer (SYH) was consulted to reach a final consensus. The preliminary screening mainly involved reviewing the titles and abstracts to determine whether they met the inclusion criteria, with potential eligible studies included. The full texts of the included studies were subsequently reviewed in detail, strictly following the inclusion and exclusion criteria. The reasons for exclusion were recorded for any studies excluded during this process. We meticulously recorded the entire screening process and created a flowchart to document it.

### 2.4 Data extraction

Two researchers (JXW and KHS) independently extracted data from the included studies. We used Excel to extract basic study characteristics, and the details of acupuncture interventions were determined based on the Standards for Reporting Interventions in Clinical Trials of Acupuncture (STRICTA) (21). After extraction, the two researchers cross-checked the results, and any discrepancies were rechecked and resolved through discussion. In most cases, available data were directly extracted from the studies. For data that could not be extracted, we contacted the original study authors to request the raw data.

### 2.5 Risk of bias assessment

Two researchers (SF and SXG) independently evaluated the risk of bias in the included studies using the Risk of Bias 2 (RoB 2) tool (22). Any discrepancies were resolved through discussion with a third reviewer (SYH). Compared to version 1.0, RoB 2.0 follows stricter methodological principles, offers clearer evaluation methods and decision rules, allows an overall assessment of the evidence quality, and reduces the subjectivity of researchers (23). Some domains yield consistent assessments across all outcomes within a study, such as Domain 1 (randomization process) and Domain 2 (deviations from intended interventions). In contrast, Domain 4 (measurement of the outcome) may vary across different outcomes. Objective outcomes, such as humoral immune factors or lung function tests, are less influenced by blinding of outcome assessors. However, for subjective outcomes such as PAQLQ, blinding both participants and outcome assessors is crucial to avoid measurement bias. The bias risk assessment tool recommended by the Cochrane Collaboration stresses the evaluation of each outcome in the included studies according to specific evaluation criteria (24). Therefore, we separately assessed the risk of bias for subjective and objective outcomes in this study.

### 2.6 Quality assessment of evidence

Two researchers (SF and XJL)assessed the quality of evidence using the Grading of Recommendations Assessment, Development, and Evaluation (GRADE) system (25, 26). RCTs were initially rated as high-quality evidence and downgrading decisions were made based on five reasons to determine the final quality rating. Disagreements regarding downgrading were resolved through consultation with a third reviewer (SYH).

### 2.7 Data synthesis

A qualitative description was provided for all included studies. We conducted the meta-analysis using R software version 4.4.2 when two or more studies were reporting the same outcome. Mean values and 95% confidence intervals were used to represent continuous data, and the difference in continuous data before and after treatment was calculated for meta-analysis. Risk ratios (RR) and 95% confidence intervals were used to represent dichotomous data. When I^2^ is less than 30%, a fixed-effect model was used to combine the data; when I^2^ is greater than 30%, a random-effects model was used. This study followed the recommendations from the Cochrane Library for systematic reviews, presenting the bias risk assessment along with the results of each study included in the meta-analysis in the forest plot (24). If more than 3 studies were included, subgroup analysis was planned to identify the sources of heterogeneity, with subgroups based on different acupuncture types and clinical phenotypes. If no source of heterogeneity was identified in the subgroup analysis, or if subgroup analysis could not be conducted, a sensitivity analysis was performed to evaluate the stability of the findings, provided that at least 3 studies were included. When only 2 studies were included, the pooling model would be modified for sensitivity analysis. For each outcome, Egger’s test was used to detect publication bias if the number of studies was greater than 4. Visual inspection of the funnel plot was added when more than 10 studies were included. If publication bias was detected, the trim-and-fill method was used to adjust the pooled results and evaluate the impact of publication bias on the combined outcome.

## 3 Results

### 3.1 Study selection

**Figure 1:**
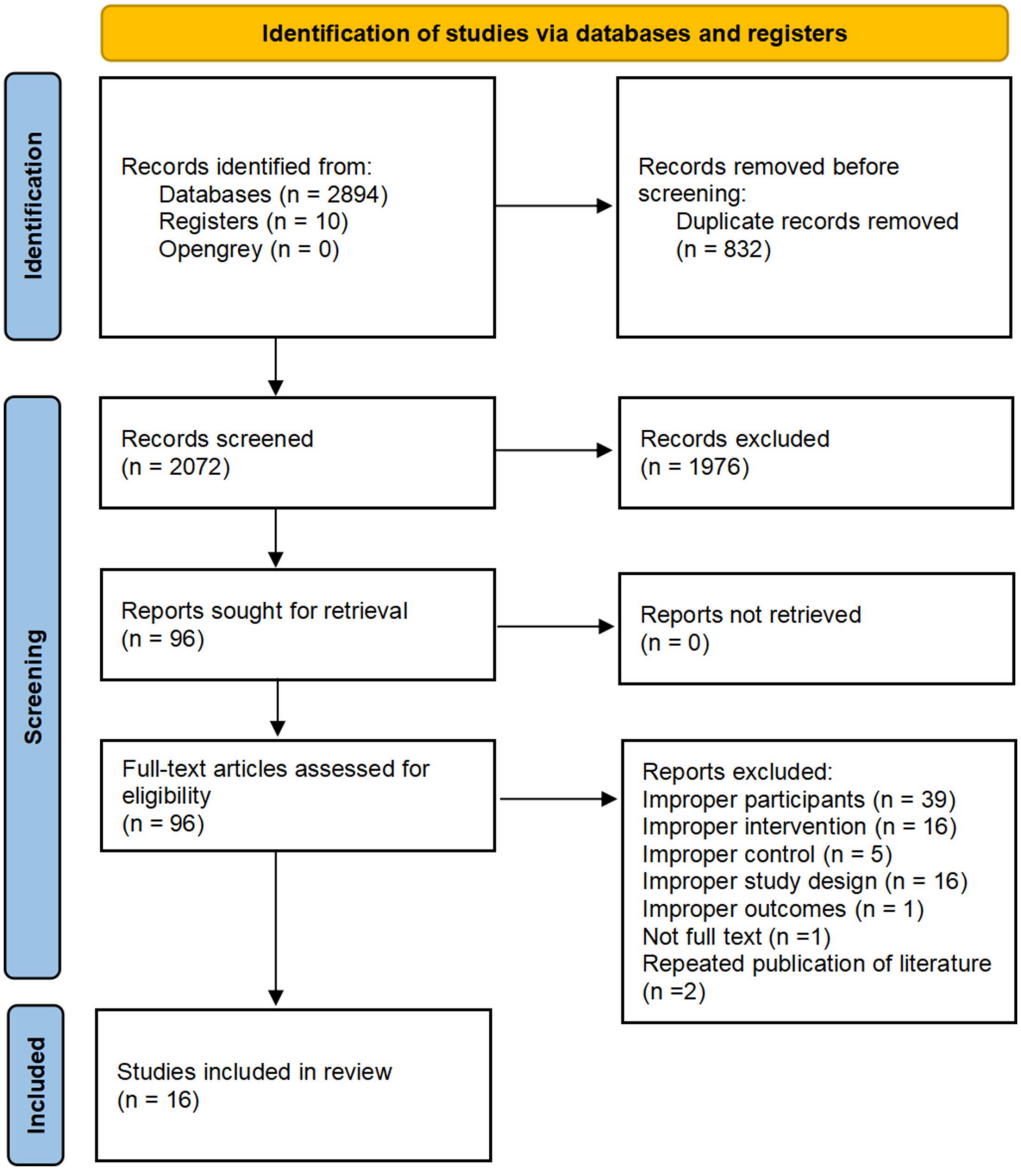
PRISMA flow chart.

A total of 2,904 studies were identified through searches of databases and trial registries, and 2,072 remained after removing duplicates using EndNote X20. After screening titles and abstracts, 1,976 irrelevant studies were excluded. A further 80 studies were excluded after full-text review. The excluded studies, along with the reasons for their exclusion, are presented in Supplementary File 2. Ultimately, 16 studies (27–42) were included for quantitative analysis. The study selection process is illustrated in Figure 1.

### 3.2 Study characteristics

**Figure 2:**
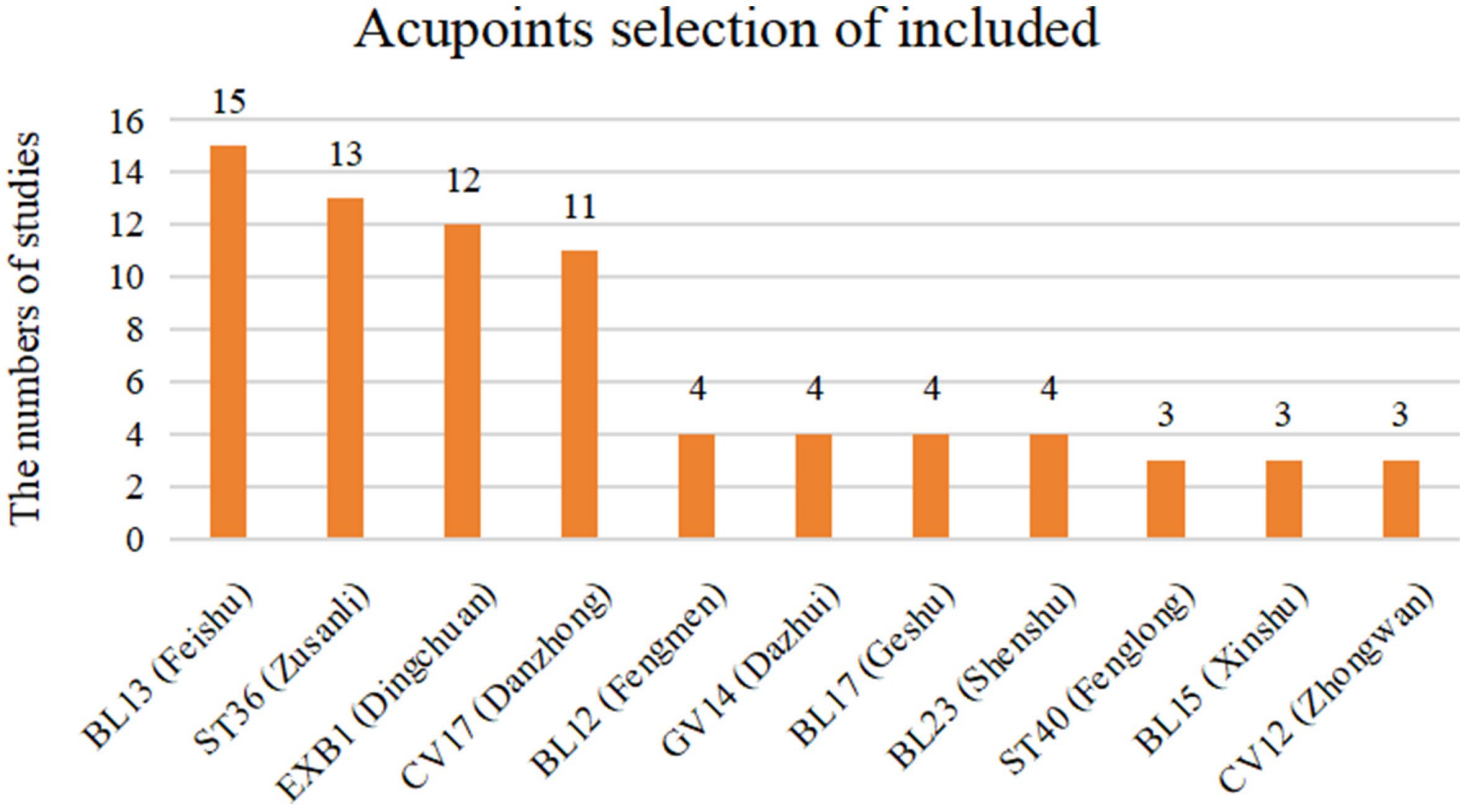
Acupoints selection of included RCTs.

These studies were conducted in China, Germany, and Saudi Arabia and were published between 2010 and 2023. Fourteen studies were published in Chinese (27, 28, 30–40, 42) and two in English (29, 41). A total of 16 RCTs were included in this study, and data from 1,675 pediatric participants were analyzed quantitatively. Among them, 839 were in the intervention group and 836 in the control group. All studies adopted a parallel, load design. 7 studies (27–29, 39–42) focused on children diagnosed with CA, while 9 studies (30–38) included children with CVA. In all studies, the intervention group received acupuncture therapies in addition to ST. 12 studies (27, 28, 30–38, 40) employed ACE, 3 studies (39, 41, 42) used MA, and 1 study (29) applied transcutaneous electrical acupoint stimulation (TEAS). Only 1 study (29) used breathing training as the control intervention, while all other studies used pharmacological treatments as controls. The duration of intervention ranged from 4 weeks to 3 months, with a 3-month treatment cycle being the most common. Only 1 study (38) reported adverse events. All included studies were analyzed, with missing data primarily handled by deletion. The demographic characteristics, outcome indicators, and intervention frequency of each study are summarized in Table 1. All studies reported the acupuncture points used, which are detailed in Figure 2. The four most frequently used acupoints were BL13 (27–42), ST36 (27, 28, 30–38, 40, 42), EX-B1 (27–34, 37–40), and CV17 (27, 28, 30–35, 37, 38, 41). This is highly consistent with previous findings on acupoint selection for asthma (43).

**Table 1:** Characteristics of included RCTs.

Included study	Sample size (E/C)	Age (E/C)	Gender (M/F)	Phenotypes	Experimental group	Acupoints	Needle specifications	Frequency	Control group	Specifications	Frequency	Duration	Outcomes
Jiang P et al. (2023) (27)	97/95	E:7.06 ± 1.05 C:7.28 ± 1.08	NR	CA	ACE plus C	EX-B1, BL13, ST36, CV17	4–0 chromic catgut 0.2–0.3	2 sessions/month 15 days/session	Salmeterol Xinafoate and Fluticasone Propionate Powder for Inhalation	150 μg	3 months 2 sessions/day	3 months	Efficacy rate, TCM symptom score, PEF, FEV1(L), FVC(L), FEV1/FVC, IgA, IgG, IgE, EOS
Yang YF et al. (2021) (28)	51/53	E:9 ± 2 C:9 ± 2	E:29/22 C:31/22	CA	ACE plus C	EX-B1, BL13, ST36, CV17	4–0 chromic catgut 0.2–0.3	2 sessions/month 15 days/session	Fluticasone Propionate Inhaled Aerosol	125 μg	2 sessions/day	3 months	Efficacy rate, TCM symptom score, PEF, FEV1pred%, MEF25%, MEF50%, MEF75%, IgA, IgE
Elnaggar et al. (2021) (29)	14/15	E:12.6 ± 1.6 C:13.4 ± 2.3	NR	CA	TEAS plus C	EX-B1	frequency: 2 Hz, pulse-width: 200 μs	3 sessions/week 40 min/session	breathing re-training	NR	NR	8 weeks	FEV1pred%, FVCpred%, FEV1/FVC, PAQLQ
Zhang HB (2020) (30)	38/38	E:9.04 ± 1.43 C:8.94 ± 1.32	E:19/19 C:19/19	CVA	ACE plus C	CV17, BL13, EX-B1, ST36	3–0 chromic catgut 0.5–0.8	NR	Montelukast Sodium Chewable Tablets	5 mg	1 session/day	3 months	Efficacy rate, cough symptom score, recurrence rate, IgA, IgE
Li FC (2020) (31)	30/30	E:10.2 C:10.5	E:16/14 C:17/13	CVA	ACE plus C	BL13, CV17, EX-B1, ST36	chromic catgut 0.2–0.3	2 sessions/first month 15 days/session 1 session/following 2 months 30 days/session	Montelukast Sodium Chewable Tablets	5 mg	1 session/day	3 months	Efficacy rate, IgE, EOS
Wang EJ et al. (2017) (32)	44/43	E:10.3 ± 1.8 C:10.8 ± 1.9	E:23/21 C:21/22	CVA	ACE plus C	BL13, CV17, EX-B1, ST36	chromic catgut 0.2–0.3	2 sessions/first month 15 days/session 1 session/following 2 months 30 days/session	Montelukast Sodium Chewable Tablets	5 mg	1 session/day	3 months	Efficacy rate, FeNO, IgE, EOS
Song SY et al. (2017) (33)	48/48	E:6.34 ± 2.86 C:6.17 ± 2.53	E:28/20 C:26/22	CVA	ACE plus C	GV14, BL12, BL13, EX-B1, CV17, ST36	0–0 chromic catgut	2 sessions/first month 15 days/session 1 session/following 2 months 30 days/session	Montelukast Sodium Chewable Tablets	4 mg/5 mg	1 session/day	3 months	Efficacy rate, IgA, IgG, IgE, IgM, CD3 + T, CD4 + T, CD8 + T, CD4 + T/CD8 + T
Wang XY et al. (2017) (34)	101/105	E:10 ± 3 C:10 ± 2	E:53/48 C:60/45	CVA	ACE plus C	BL13, CV17, EX-B1, ST36	4–0 chromic catgut 0.2–0.3	2 sessions/first month 15 days/session 1 session/following 2 months 30 days/session	Montelukast Sodium Chewable Tablets	5 mg	1 session/day	3 months	Efficacy rate, cough symptom score, IgA, IgG, IgE, FEV1pred%, PEF, MEF25%, MEF50%, MEF75%
Tao BT (2017) (35)	45/45	E:5.4 ± 2.3 C:5.5 ± 2.1	E:25/20 C:23/22	CVA	ACE plus C	BL15, BL13, BL17, CV17, ST36	NR	1 session/month 30 days/session	Montelukast Sodium Chewable Tablets	4 mg/5 mg	1 session/day	3 months	Efficacy rate, IFN-γ, IL-4, IL-5
Jin YJ et al. (2015) (36)	36/38	E:3.13 ± 0.96 C:2.98 ± 1.03	E:20/16 C:19/19	CVA	ACE plus C	Main Points: GV14, BL12, BL13, BL23, ST36	4–0 chromic catgut 0.4–0.5	1 session/month 30 days/session	Montelukast Sodium Chewable Tablets	4 mg/5 mg	1 session/day	3 months	Efficacy rate, IFN-γ, IL-4, IL-5
Liu J et al. (2015) (37)	60/60	E:8.2 C:8	E:33/27 C:34/26	CVA	ACE plus C	BL13, BL15, BL17, CV17, EX-B1, ST36	NR	2 sessions/first month 15 days/session 1 session/following 2 months 30 days/session	Provide compound ipratropium bromide and budesonide suspension as needed.	4 mg/5 mg	1 session/day	3 months	Efficacy rate, IgA, IgG, IgM, FEF25%, FEF50%, FEF75%, FEF25-75%
Lu B (2015) (38)	31/29	E:9.84 ± 2.55 C:10.22 ± 2.31	E:19/12 C:13/16	CVA	ACE plus C	BL13, CV17, EX-B1, ST36	3–0 chromic catgut 0.5–0.8	2 sessions/first month 15 days/session 1 session/following 2 months 30 days/session	Montelukast Sodium Chewable Tablets	5 mg	1 session/day	3 months	Efficacy rate, recurrence rate, cough symptom score, IgA, IgE, safety evaluation
Lin JX (2013) (39)	58/50	Total:4.97 ± 3.45	Total:68/40	CA	MA plus C	BL13, BL20, BL23, GV14, EX-B1, BL12	NR	1 session/day 20 min/session	Provide aminophylline and corticosteroids as needed.	NR	NR	3 months	Recurrence rate, IgE
Han X et al. (2012) (40)	30/30	E:4.13 ± 1.93 C:4.96 ± 1.32	E:16/14 C:17/13	CA	ACE plus C	Main Points: GV14, EX-B1, BL13, BL23, ST36, ST40.	chromic catgut 0.5–0.6	1 session/month 30 days/session	Montelukast Sodium Chewable Tablets	4 mg/5 mg	1 session/day	3 months	Efficacy rate, IgE, IL-4
Scheewe S et al. (2011) (41)	46/47	E: male 14.6 ± 1.8; female 15 ± 2.1 C: male 13.7 ± 1.2; female 16.2 ± 1.7	E:26/20 C:25/22	CA	MA plus C	Main Points: BL13, CV17, LU7	0.18 × 13 mm; 0.3 × 30 mm	3 sessions/week 30 min/session	Use SABA as needed	NR	NR	4 weeks	FEV1/FVC, MEF50, DeltaFEV1, PEF variability, STAIC, PAQLQ
Lan CY (2010) (42)	110/110	E:7.1 ± 1.6 C:7.2 ± 1.5	E:52/58 C:53/57	CA	MA plus C	Main Points: BL13, BL20, ST36, CV12, SP3	NR	NR 30 min/session	Provide ketotifen, procaterol, and beclomethasone inhaler as needed.	0.5 mg/1 mg 1.25 mg/kg 50 μg	NR	1 month	Efficacy rate, FEV1(L), FEV1pred%, PEFpred%, daytime symptom score, nighttime symptom score

E, experimental group; C, control group; M, male; F, female; CA, classical asthma; CVA, cough variant asthma; NR, not reported; MA, manual acupuncture; ACE, acupoint catgut embedding; TEAS, transcutaneous electrical acupoint stimulation.

### 3.3 Risk of bias

**Figure 3:**
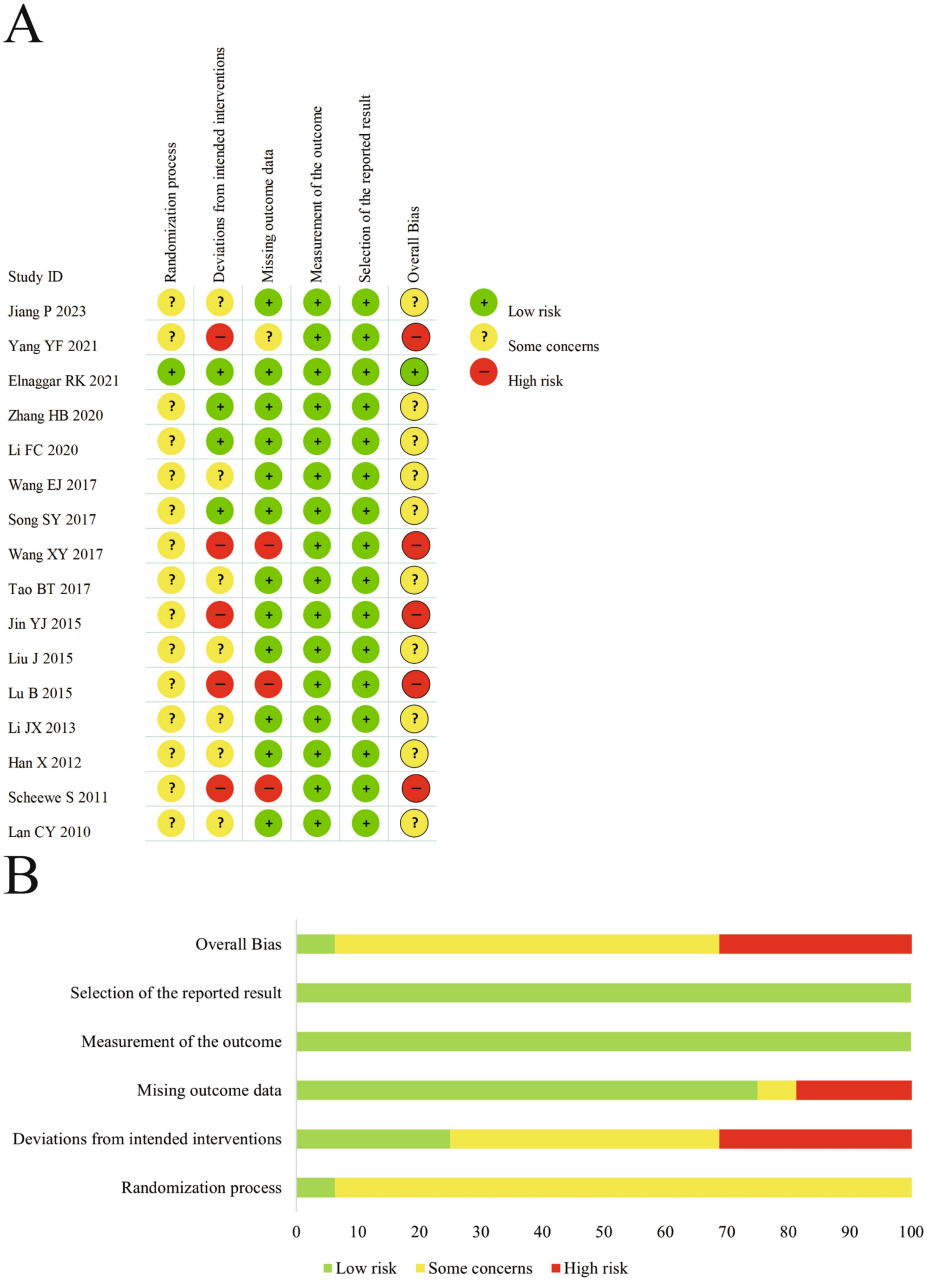
**(A)** The risk of bias graph for objective outcomes. **(B)** The summary of risk of bias for objective outcomes.

**Figure 4:**
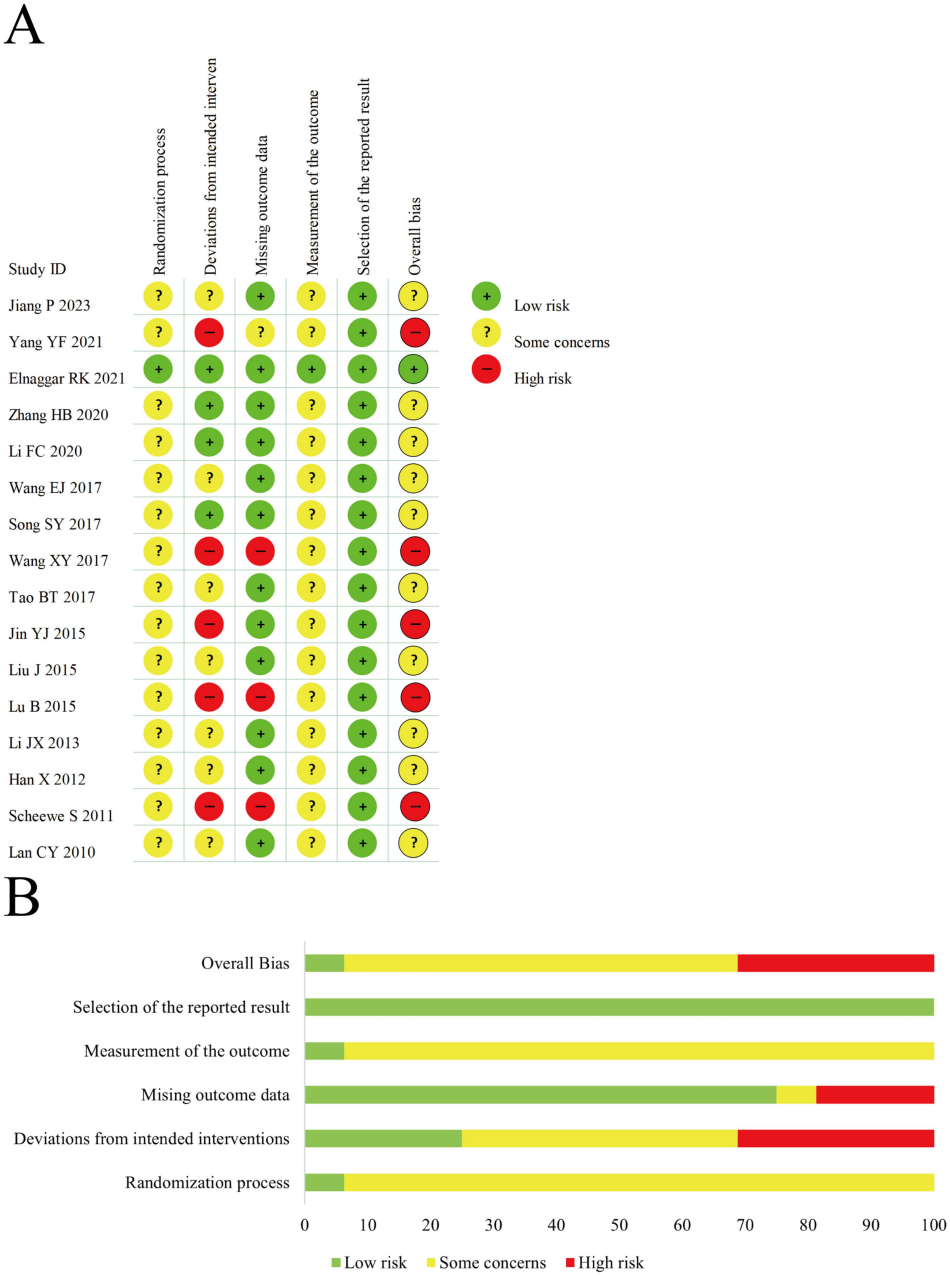
**(A)** The risk of bias graph for subjective outcomes. **(B)** The summary of risk of bias for subjective outcomes.

We assessed the risk of bias in all included studies; objective outcomes are presented in Figure 3 and subjective outcomes in Figure 4. In Domain 1 (randomization process), 15 trials (27, 28, 30–42) were judged as having some concerns, and 1 trial (29) was assessed as low risk. In Domain 2 (deviations from intended interventions), 5 studies (28, 34, 36, 38, 41) were at high risk, 4 (29–31, 33) at low risk, and 7 (27, 32, 35, 37, 39, 40, 42) had some concerns. In Domain 3 (missing outcome data), 3 trials (34, 38, 41) were judged to be at high risk, 12 (27, 29–33, 35–37, 39, 40, 42) were at low risk, and 1 (28) had some concerns. In Domain 4 (measurement of the outcome), all trials were assessed as low risk for serum immune and inflammatory biomarkers and pulmonary function parameters. However, for subjective outcomes such as efficacy rate and PAQLQ, all but 1 study (29) were rated as having some concerns. In Domain 5 (selection of the reported result), all studies were rated as low risk. In terms of overall risk of bias, only 1 study (29) was at low risk, 5 were at high risk, and 10 were judged to have some concerns.

### 3.4 Meta-analysis

#### 3.4.1 The percent predicted values of forced expiratory volume in 1 second

**Figure 5:**
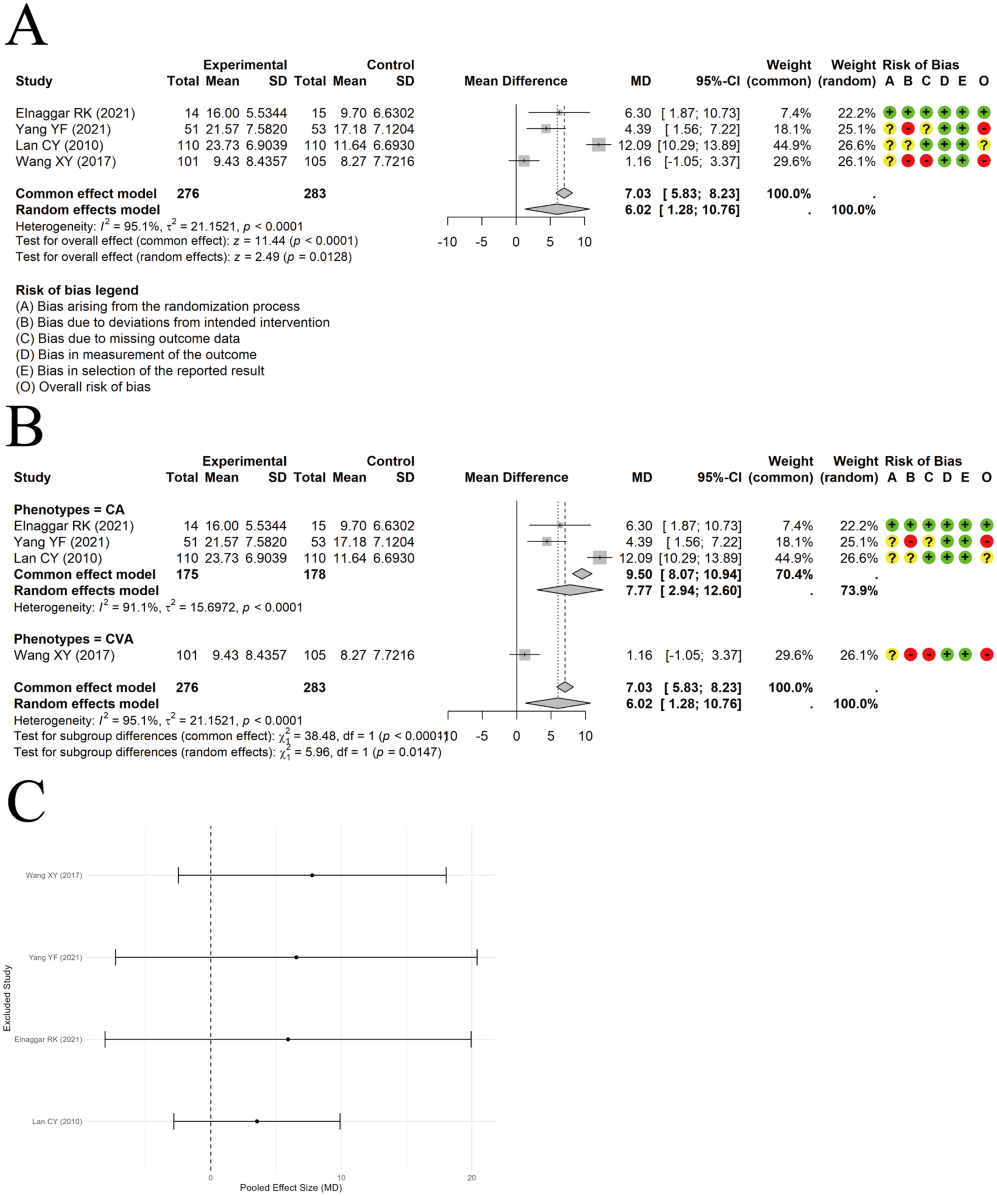
The comparison between acupuncture plus ST and ST: FEV1pred%. **(A)** Forest plot. **(B)** Subgroup analysis. **(C)** Sensitivity analysis.

A random-effects model was used to pool the percent predicted values of forced expiratory volume in 1 second (FEV1pred%) data from 4 trials. The results indicated that acupuncture combined with ST improved FEV1pred% compared to ST alone [MD = 6.02, 95% CI (1.28, 10.76), *p* = 0.0128] (Figure 5A). However, significant heterogeneity was observed (I^2^ = 95.1%, *p* < 0.00001). Subgroup analysis by clinical phenotype did not significantly reduce heterogeneity (Figure 5B), suggesting that clinical phenotype may not be the source of heterogeneity. Sensitivity analysis using the leave-one-out method revealed that the results were not stable (Figure 5C). The current evidence may not support the conclusion that acupuncture combined with standard therapy improves FEV1pred%. Egger’s test showed no evidence of publication bias (t = −0.61, df = 2, *p* = 0.6014). However, the number of included studies was less than 10, resulting in low test power.

#### 3.4.2 Forced expiratory volume in 1 second/forced vital capacity

**Figure 6:**
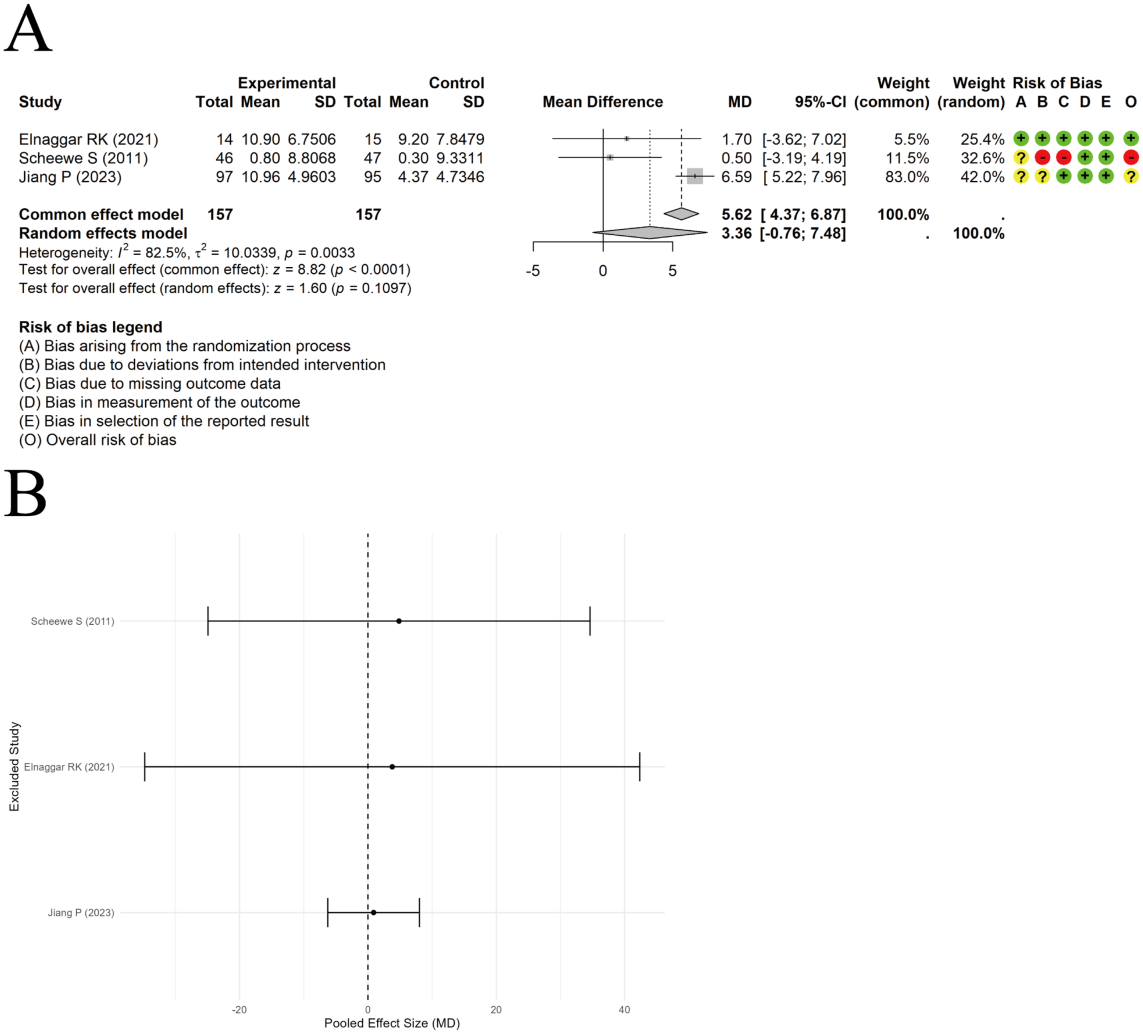
The comparison between acupuncture plus ST and ST: FEV1/FVC. **(A)** Forest plot. **(B)** Sensitivity analysis.

A random-effects model was used to pool the Forced expiratory volume in 1 second/forced vital capacity (FEV1/FVC) data from 3 trials. The results indicated no significant difference between acupuncture combined with ST and ST alone [MD = 3.36, 95% CI (−0.76, 7.48), *p* = 0.1097] (Figure 6A), with substantial heterogeneity observed among studies (I^2^ = 82.5%, *p* < 0.00001). Sensitivity analysis suggested that the results were not influenced by any single study (Figure 6B).

#### 3.4.3 IgE

**Figure 7:**
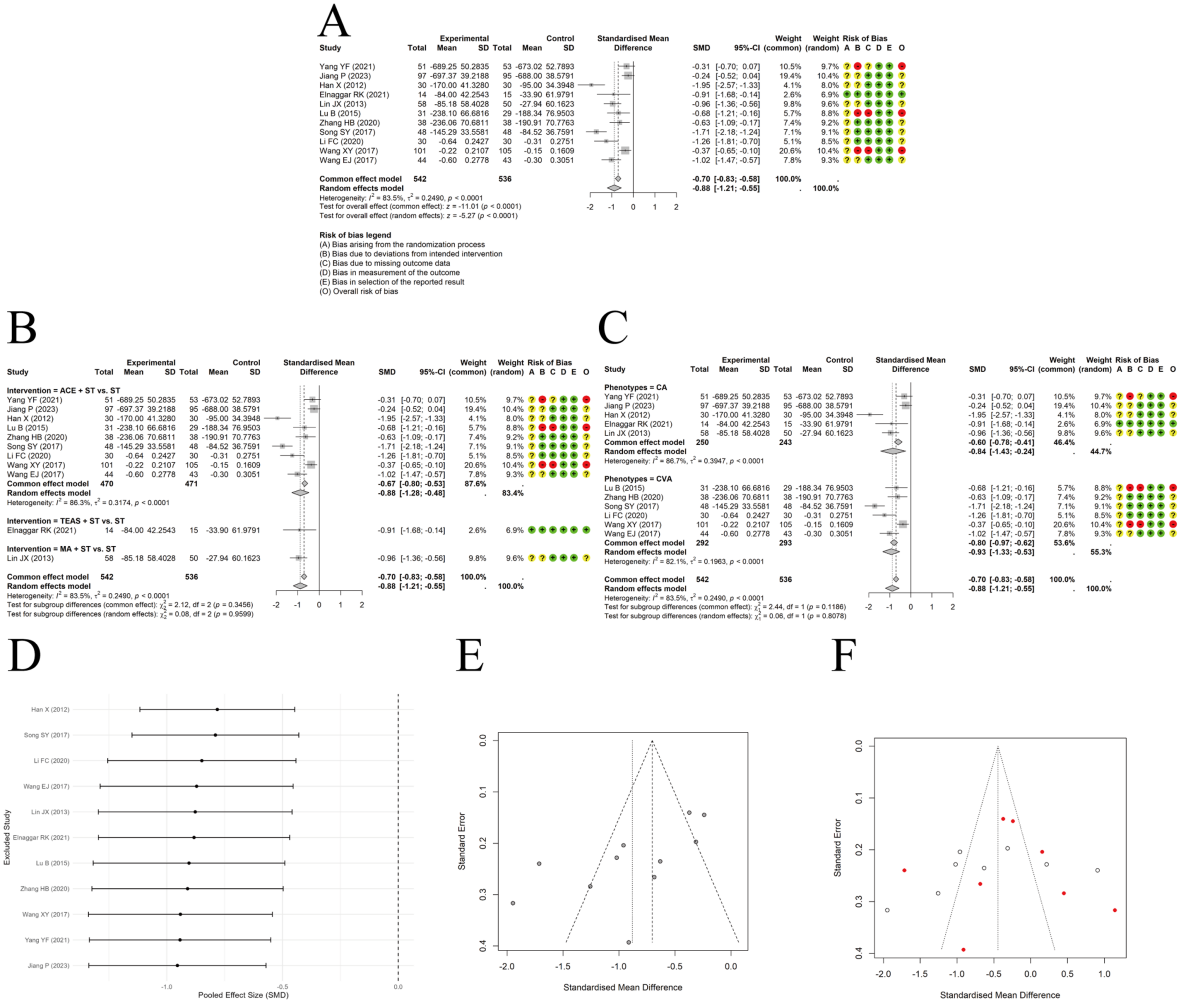
The comparison between acupuncture plus ST and ST: IgE. **(A)** Forest plot. **(B)** Subgroup analyses stratified by type of acupuncture. **(C)** Subgroup analyses stratified by clinical phenotypes. **(D)** Sensitivity analysis. **(E)** Funnel plots. **(F)** Funnel plot after trim-and-fill method.

A random-effects model was used to pool the IgE outcomes reported in 11 trials. The results showed that acupuncture combined with ST significantly reduced IgE levels compared to ST alone, with a large effect size [SMD = −0.88, 95% CI (−1.21, −0.55), *p* < 0.0001] (Figure 7A). However, substantial heterogeneity was observed among the included studies (I^2^ = 83.5%, p < 0.00001). Subgroup analyses did not identify clinical phenotype or type of acupuncture as the source of heterogeneity (Figures 7B,C). Sensitivity analysis by sequentially removing each study indicated the results were relatively robust (Figure 7D). Funnel plot asymmetry was observed (Figure 7E), and Egger’s test suggested the presence of publication bias (t = −3.11, df = 9, *p*-value = 0.0125). A trim-and-fill analysis was conducted to assess the robustness of the pooled effect size, estimating 8 potentially missing studies. The adjusted effect size was [SMD = −0.423, 95% CI (−0.312, 7.480)] (Figure 7F), still indicating a small but statistically significant effect. Due to inconsistencies in measurement units across studies, the standardized mean difference was used for synthesis.

#### 3.4.4 IgA

**Figure 8:**
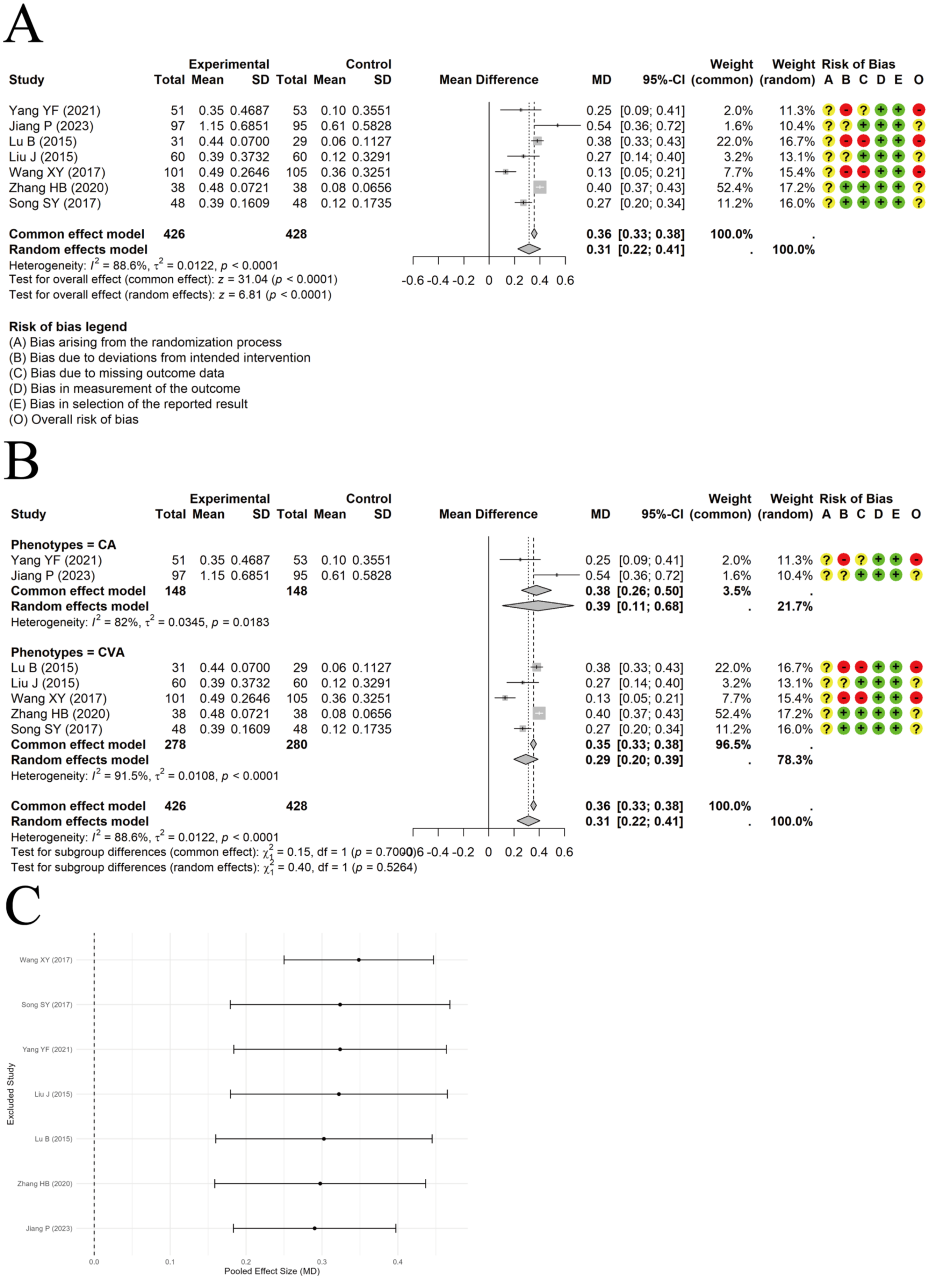
The comparison between acupuncture plus ST and ST: IgA. **(A)** Forest plot. **(B)** Subgroup analysis. **(C)** Sensitivity analysis.

A random-effects meta-analysis was conducted on the IgA data extracted from 7 included trials. The results indicated that acupuncture combined with ST significantly increased IgA levels compared to ST alone [MD = 0.31, 95% CI (0.22, 0.41), *p* < 0.0001] (Figure 8A). Substantial heterogeneity was observed across studies (I^2^ = 88.6%, *p* < 0.0001). Subgroup analyses suggested that clinical asthma phenotypes or acupuncture types were unlikely to explain the heterogeneity (Figure 8B). Sensitivity analysis by excluding studies one at a time showed the results were relatively robust (Figure 8C). Egger’s test indicated no significant publication bias (t = −1.18, df = 5, *p*-value = 0.2902).

#### 3.4.5 IgG

**Figure 9:**
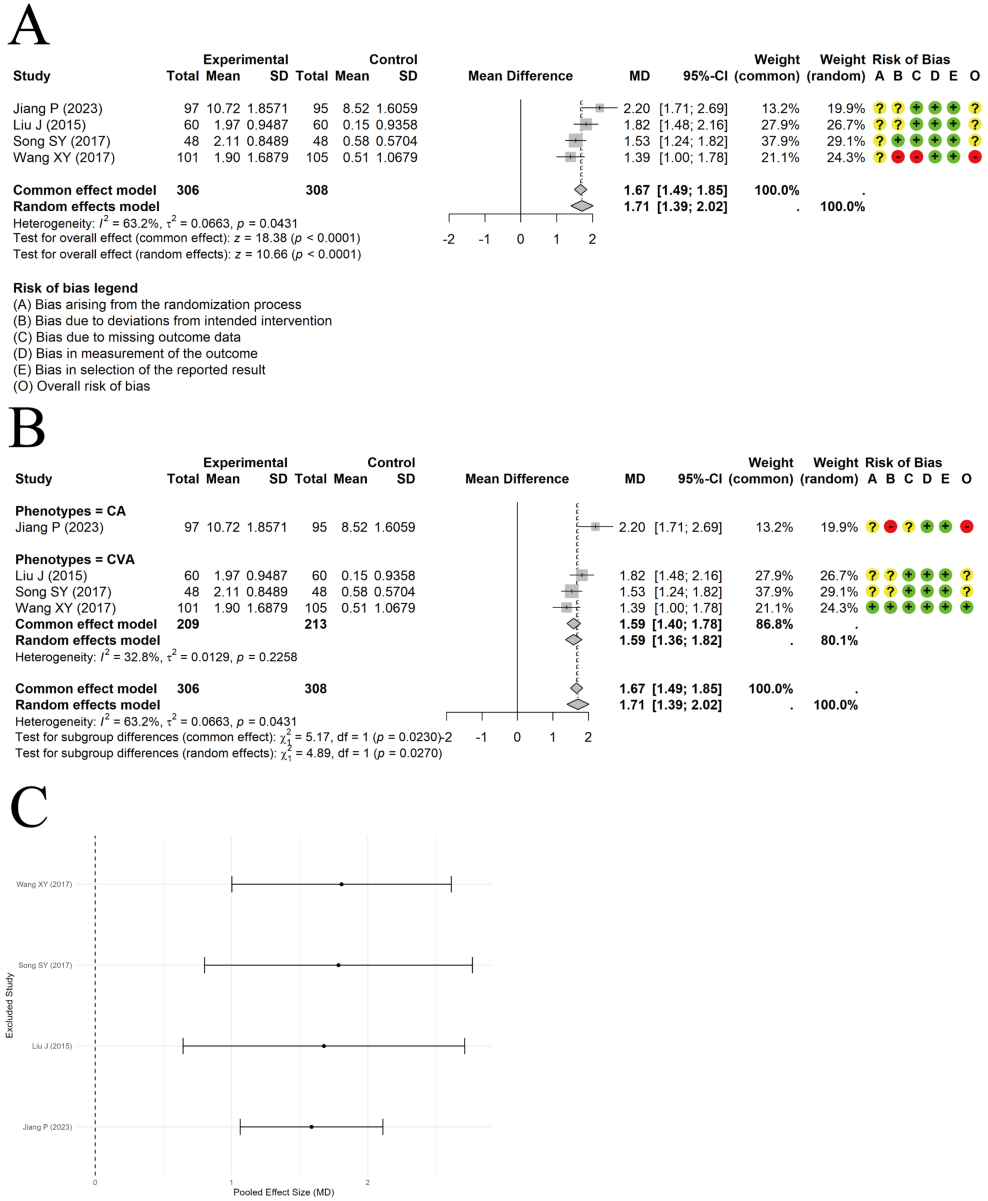
The comparison between acupuncture plus ST and ST: IgG. **(A)** Forest plot. **(B)** Subgroup analysis. **(C)** Sensitivity analysis.

A random-effects model was used to pool data from 4 trials reporting IgG levels, and the results demonstrated a significant difference between acupuncture combined with ST and ST alone in increasing IgG levels [MD = 1.71, 95% CI (1.39, 2.02), *p* < 0.0001] (Figure 9A). Moderate heterogeneity was observed among the included studies (I^2^ = 63.2%, *p* = 0.0431). Although heterogeneity decreased in the subgroup analysis, unequal sample sizes between subgroups prevented confirmation of clinical phenotype as a source of heterogeneity (Figure 9B). Sensitivity analysis indicated that the results were relatively robust (Figure 9C). Egger’s test indicated no significant publication bias (t = 1.07, df = 2, p-value = 0.3979).

#### 3.4.6 PEF

**Figure 10:**
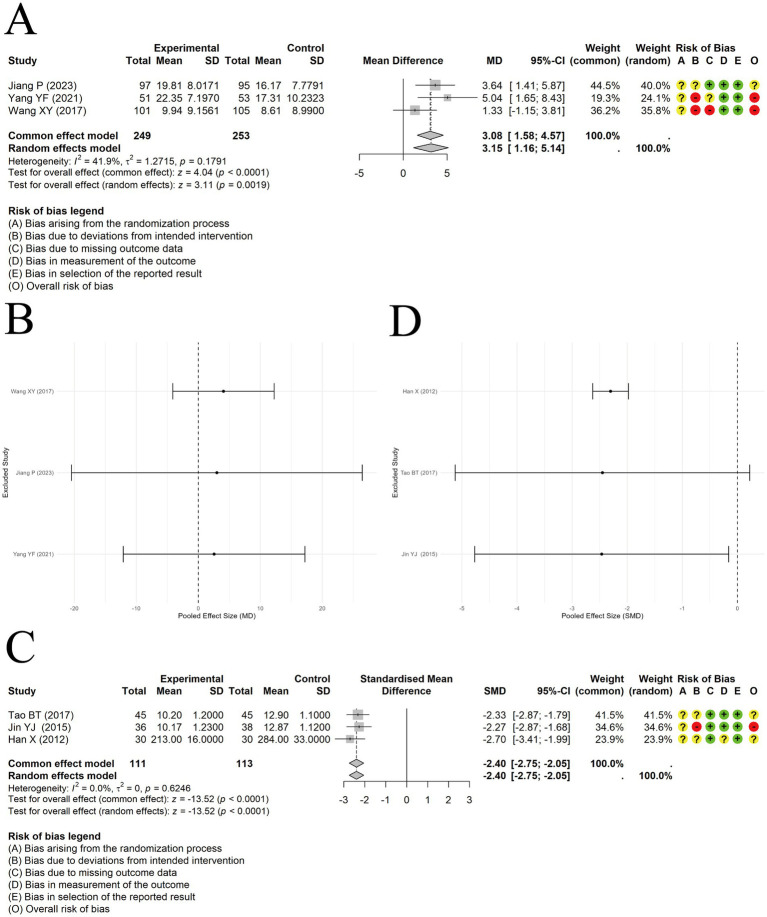
The comparison between acupuncture plus ST and ST: PEF. **(A)** Forest plot. **(B)** Sensitivity analysis. The comparison between acupuncture plus ST and ST: IL-4. **(C)** Forest plot. **(D)** Sensitivity analysis.

A random-effects approach was applied to integrate findings on PEF from 3 trials. The results showed a significant difference in improving PEF between acupuncture combined with ST and ST alone [MD = 3.15, 95% CI (1.16, 5.14), *p* = 0.0019] (Figure 10A). Sensitivity analysis revealed that the results were not robust (Figure 10B).

#### 3.4.7 IL-4

A fixed-effect model was applied to synthesize IL-4 levels from 3 trials. A marked reduction in IL-4 levels was observed in the acupuncture plus ST group relative to ST alone, with a large effect size [SMD = -2.40, 95% CI (−2.75, −2.05), *p* < 0.0001] (Figure 10C). There was no heterogeneity among the included studies (I^2^ = 0). However, during IL-4 data extraction, 1 study lacked baseline data, so only post-treatment IL-4 levels were pooled, and pre-post differences could not be calculated. Therefore, we have limited confidence in this conclusion. Sensitivity analysis also indicated that the results were not robust (Figure 10D).

#### 3.4.8 EOS

**Figure 11:**
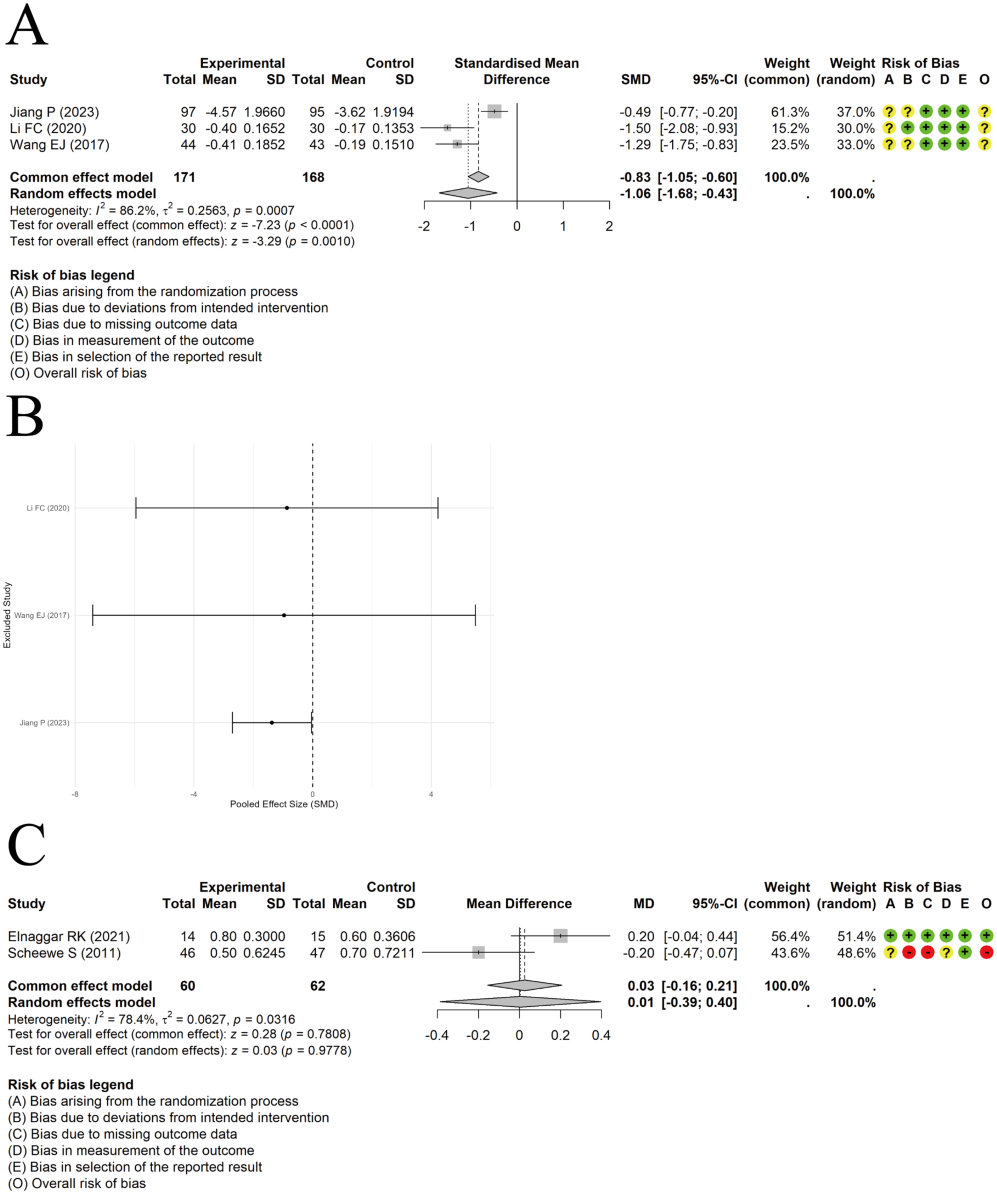
The comparison between acupuncture plus ST and ST: EOS. **(A)** Forest plot. **(B)** Sensitivity analysis. The comparison between acupuncture plus ST and ST: PAQLQ. **(C)** Forest plot and Sensitivity analysis.

Acupuncture in conjunction with ST significantly lowered EOS levels compared to ST alone, with a meaningful effect size [MD = −1.06, 95% CI (−1.68, −0.43), *p* = 0.0010] (Figure 11A). Substantial heterogeneity was observed among the studies (I^2^ = 86.2%, *p* = 0.0007). Sensitivity analysis excluding individual studies revealed that the results lacked stability (Figure 11B).

#### 3.4.9 PAQLQ

A random-effects model was employed to synthesize results from a pair of studies evaluating PAQLQ scores. The results showed no significant difference in quality-of-life improvement between acupuncture plus ST and ST alone [MD = 0.01, 95% CI (−0.39, 0.40), *p* = 0.9778] (Figure 11C). Substantial heterogeneity was present among the studies (I^2^ = 78.4%, *p* = 0.0316). When switching to a fixed-effects model, the difference between the two groups remained non-significant [MD = 0.03, 95% CI (−0.16, 0.21), *p* = 0.7808].

### 3.5 Quality of evidence

Due to the risk of bias and inconsistency in the included studies, the quality of evidence for most outcomes of interest has been rated as low, the detailed explanations of the evidence grading results and reasons for downgrading are presented in Table 2. It is noteworthy that we did not downgrade for publication bias in outcomes with fewer than 10 studies, as the power of Egger’s test is reduced in such cases, and downgrading for publication bias should be conservative. Therefore, caution is needed when interpreting the results.

**Table 2:** GRADE of evidence of outcomes of the included studies.

Outcomes	Number of studies (total sample)	Pooled effect size	Certainty assessment					Quality of evidence
Acupuncture plus ST vs. ST			Limitations	Inconsistency	Indirectness	Imprecision	Publication bias	
FEV1pred%	4 (559)	MD = 6.02, 95% CI (1.28, 10.76)	−1^①^	−1^②^	0	0	0	Low⊕⊕⊖⊖
FEV1/FVC	3 (314)	MD = 3.36, 95% CI (−0.76, 7.48)	−1^①^	−1^②^	0	−1^③^	0	Very low⊕⊖⊖⊖
IgE	11 (1078)	SMD = −0.423, 95% CI (−0.312, 7.48)	−1^①^	−1^②^	0	0	−1^④^	Very low⊕⊖⊖⊖
IgA	7 (854)	MD = 0.31, 95% CI (0.22, 0.41)	−1^①^	−1^②^	0	0	0	Low⊕⊕⊖⊖
IgG	4 (614)	MD = 1.71, 95% CI (1.39, 2.02)	−1^①^	−1^②^	0	0	0	Low⊕⊕⊖⊖
PEF	3 (502)	MD = 3.15, 95% CI (1.16, 5.14)	−1^①^	−1^②^	0	0	0	Low⊕⊕⊖⊖
IL-4	3 (224)	SMD = −2.40, 95% CI (−2.75, −2.05)	−1^①^	0^②^	0	−1^③^	0	Low⊕⊕⊖⊖
EOS	3 (339)	MD = −1.06, 95% CI (−1.68, −0.43)	−1^①^	−1^②^	0	−1^③^	0	Very low⊕⊖⊖⊖
PAQLQ	2 (122)	MD = 0.01, 95% CI (−0.39, 0.40)	−1^①^	−1^②^	0	−1^③^	0	Very low⊕⊖⊖⊖

SMD, standardized mean difference; MD, mean difference; CI, confidence interval; ST, standard treatment; ①, The design of the study with a large bias in random, distributive hiding or blind; ②, The confidence interval overlaps less, the heterogeneity test P is Critically small, and the I^2^ > 50%; ③, The Confidence interval is not narrow enough, and *n* < 500; ④, Egger test detected the presence of publication bias. High-quality evidence indicates that further research is very unlikely to change confidence in the estimate of the effect. Moderate-quality evidence suggests that further research may have an important impact on confidence in the estimate and may change the estimate. Low-quality evidence implies that further research is likely to have an important impact on the confidence in the estimate and is likely to change the estimate. Very low-quality evidence reflects great uncertainty about the estimate, and the true effect is likely to be substantially different.

## 4 Discussion

### 4.1 Summary of results

This study has conducted a meta-analysis of 16 studies involving 1,675 children to evaluate the efficacy and safety of acupuncture as an adjunct to ST. The results have indicated insufficient confidence in the effect of acupuncture on improving FEV1pred%, the primary outcome. This conclusion has been consistent with that of Liu et al. (17). No significant differences have been observed in FEV1/FVC and quality of life. However, this study has found that adjunctive acupuncture reduced serum IgE levels and increased serum IgA and IgG levels. Although acupuncture demonstrated substantial regulatory effects on inflammatory and immune markers such as IgE, IgA, and IgG, the corresponding improvements in key lung function parameters (FEV1pred% and PEF) were relatively modest. This discrepancy may be attributed to the complex and indirect relationship between immune modulation and pulmonary function parameters. Pulmonary function indices reflect integrated airway dynamics and structural changes, which may require longer intervention durations or more profound remodeling to manifest measurable change. In pediatric asthma, inflammation, and airway remodeling may occur at an early stage, even before measurable lung function impairment becomes apparent. Saglani et al. (44) demonstrated that preschool wheezers can exhibit marked subepithelial fibrosis and smooth muscle thickening in the absence of lung volume abnormalities. Pulmonary function parameters may not immediately reflect the extent of underlying structural and inflammatory changes, particularly in early or mild disease. Therefore, the immunological improvements observed in our study may not directly translate into substantial changes in pulmonary function, especially within the relatively short treatment duration. Regarding safety, only 1 study has reported that acupoint catgut embedding might cause mild itching or slight erythema in a small number of children. Overall, the included studies have exhibited unclear risk of bias and high heterogeneity, resulting in predominantly low-quality evidence, necessitating further research to confirm these findings.

### 4.2 Clinical interpretation

CA and CVA have been identified as the most common phenotypes in pediatric asthma. The GINA 2024 guidelines have stratified standard asthma treatments by age group. For children aged 5 years and younger, trial use of low-dose ICS has been recommended when asthma is suspected, with treatment response evaluated within 3 months. Due to the side effects of ICS, the benefit–risk ratio in younger children has remained uncertain. For this age group, certain green and effective complementary and alternative therapies have shown notable advantages. In 1979, asthma was included by the World Health Organization (WHO) as an indication for acupuncture use. Acupuncture, as a common complementary and alternative therapy, has offered specific advantages for the pediatric population. However, MA or EA has sometimes provoked fear among children. The subsequently developed ACE has provided prolonged stimulation with fewer sessions, improving compliance in children. An increasing number of clinical trials have investigated the efficacy and safety of acupuncture in pediatric bronchial asthma. Karlson et al. (45) have reported that acupuncture reduced the use of ICS and β2-agonists in children compared to sham acupuncture. A meta-analysis of LA for childhood asthma has found no convincing evidence of its efficacy (18). A systematic review of acupuncture in children has suggested possible benefits on PEF and diurnal variation in PEF among asthmatic children (17). It is noteworthy that this review included crossover-designed studies, resulting in inconsistent data formats that precluded meta-analysis, and findings were presented qualitatively. Unlike previous studies, this study has been the first to conduct a systematic review and meta-analysis of parallel and load design RCTs evaluating acupuncture and related therapies in pediatric bronchial asthma.

### 4.3 Mechanisms of acupuncture in asthma

Acupuncture treatment protocols for the same disease have typically included certain fixed acupoints, with a few additional points individualized based on syndrome differentiation. Among all studies, the most frequently used acupoints have been BL13, ST36, EXB1, and CV17. Nurwati et al. (46) have demonstrated that acupuncture at BL13 and ST36 reduced neutrophil and eosinophil counts in asthmatic mice. This finding has been consistent with our results showing that acupuncture lowered IgE and IL-4 levels. The mechanism may involve suppression of IgE secretion by plasma cells, resulting in decreased eosinophil activation and reduced release of cytokines such as IL-4. Yu et al. (47) have found that acupoint catgut embedding at BL13, EXB1, and CV17 in asthmatic rats reduced pulmonary levels of p-p38 MAPK and IL-4. Morphologically, the acupuncture group has shown decreased thickness in the bronchial wall and bronchial smooth muscle. This effect may have occurred via modulation of Th2 cell function through the p38 MAPK pathway, leading to suppressed IL-4 transcription and reduced airway inflammation. Zhao et al. (48) have found that acupuncture at GV14, BL13, and ST36 in asthmatic mice reduced the expression of autophagy-related proteins in lung tissue, thereby alleviating airway remodeling. This has been accompanied by increased IFN-*γ* and decreased IL-4 and IL − 17 levels. Although experimental studies have shown that acupuncture affects airway remodeling in asthma, Clinical trials have not demonstrated significant improvements in remodeling as measured by FEV1 and PEF. Interestingly, although airway remodeling is a hallmark of asthma pathology, it does not necessarily result in immediate or measurable changes in pulmonary function parameters (e.g., FEV1/FVC), particularly in pediatric patients. Remodeling may initially involve structural changes such as increased smooth muscle mass, epithelial thickening, and subepithelial fibrosis, which do not produce airflow limitation detectable by spirometry. Children with normal lung function may still exhibit significant remodeling, indicating a dissociation between histological changes and functional impairment (44, 49). This may help explain the discrepancy between mechanistic study findings and clinical trial outcomes. Longer intervention periods and extended follow-up may be required to detect changes in pulmonary function.

### 4.4 Considerations for future research and clinical practice

The methodological quality of the included studies was not high, primarily due to the lack of allocation concealment and blinding in the randomization process, which may have led to deviations from the intended interventions. The low methodological quality undermines the credibility of the study findings, thereby affecting the overall quality of evidence.

We should identify the population that benefits most from acupuncture in pediatric asthma by setting strict inclusion and exclusion criteria in the design of explanatory randomized controlled trials. The included studies lacked detailed descriptions of acupuncture interventions, which did not conform to reporting standards for acupuncture clinical trials (21). The absence of detailed descriptions compromises researchers’ ability to evaluate intervention quality and clinical heterogeneity, while also reducing the reproducibility of the studies. Acupuncture point selection should be based on standardized protocols with individualized modifications. The control group should be selected according to asthma phenotype and disease severity. The treatment principles for pediatric asthma emphasize symptom-based management to minimize future risks. The treatment goals for classic asthma include achieving and maintaining symptom control, preserving normal activity levels, maintaining near-normal lung function, reducing the frequency of exacerbations, avoiding adverse drug reactions, and preventing asthma-related mortality. For children with CVA, improvement in cough symptoms should also be addressed. The studies we evaluated had relatively short follow-up periods, lacked sufficient data to assess the long-term efficacy of acupuncture, and reported low rates of adverse events.

The following are considerations for future research: (1) Future trials should adopt centralized randomization or use sealed opaque envelopes. The method used to generate the random sequence should also be clearly described to improve the transparency and reproducibility of the study. Moreover, the success of blinding procedures should be assessed using validated tools such as James’ or Bang’s blinding index. (2) In future explanatory RCTs, the optimal age range for participant inclusion should be determined based on pilot data or previous research findings to ensure population homogeneity. For subsequent large-scale pragmatic RCTs, stratified randomization or predefined subgroup analyses based on age groups or asthma severity (e.g., baseline FEV1pred%) should be incorporated. These strategies would help identify which pediatric subpopulations derive the most clinical benefit from acupuncture interventions and reduce the influence of heterogeneity on outcome interpretation. (3) Regardless of the type of acupuncture used, reporting should follow the CONSORT statement and STRICTA guidelines. Ideally, acupuncture should be performed by one or two qualified practitioners with over 5 years of experience. The standardized point selection protocol should be derived from expert consensus or pilot study results. Based on this standard, 1–2 individualized points can be added, consistent with the personalized treatment concept of modern medicine. (4) In explanatory RCTs, superiority tests can be conducted using sham stimulation or waitlist controls. In pragmatic RCTs, non-inferiority tests should compare acupuncture directly with first-line medications. (5) Regarding outcome measures, more terminal outcomes should be included, such as the number of children experiencing asthma exacerbations within 1 year and the proportion achieving clinical control. For surrogate outcomes, assessment tools such as the childhood ACT, asthma control questionnaire – 7 items, and test for respiratory and asthma control in kids should be used, with the selection tailored to the age of the children to evaluate asthma control. The frequency of rescue medication use can also be recorded. For CVA, attention should be paid to improvements in cough symptoms, using measures such as changes in cough symptom scores or cough relief time/rate. Improvements in small airway obstruction should also be considered, using indices such as maximal mid-expiratory flow between 25 and 75% of the FVC, forced expiratory flow at 50% of the FVC, and forced expiratory flow at 75% of the FVC. Additionally, relevant biomarker assessments can be integrated to more accurately evaluate the effects of acupuncture on airway remodeling in pediatric asthma. Future RCTs should incorporate longer follow-up periods (e.g., 6–12 months) to capture sustained effects, recurrence rates, and any delayed adverse events associated with acupuncture treatment in pediatric asthma. (6) Most of the included studies were conducted in China and involved exclusively Chinese pediatric participants. This geographical and ethnic homogeneity may limit the external validity and generalizability of the findings. Future research should aim to include more ethnically and culturally diverse populations to better evaluate the global applicability of acupuncture in pediatric asthma management.

### 4.5 Limitations of the study

This study has several limitations. Although we pre-specified subgroup analyses based on acupuncture type and clinical phenotype, they did not reduce inconsistency. In particular, the limited number of studies evaluating individual outcomes resulted in very small subgroup sizes, which constrained the exploration of heterogeneity and interpretation of results. This does not imply that acupuncture type and clinical phenotype are not potential sources of heterogeneity. Children with asthma in different age groups exhibit distinct clinical manifestations, and the dosage of ST varies accordingly; therefore, clinical and methodological heterogeneity may stem from age-related differences in asthma phenotypes and variations in the dose and duration of standard pharmacological treatments. Unfortunately, most of the included trials did not provide stratified data, limiting our ability to perform subgroup analyses based on age or dosage. Moreover, the diversity in acupuncture point selection may have contributed to clinical heterogeneity. Methodological heterogeneity may have arisen from the generally low methodological quality of the included studies. Although the predefined subgroup analyses failed to resolve the observed heterogeneity, we conducted sensitivity analyses to assess the robustness of the pooled estimates. For outcomes with high I^2^ values, sequential exclusion of individual studies helped identify outlier effects and ensured that any single study did not unduly influence the conclusions. This analytical approach partially compensated for the inability to fully identify the sources of heterogeneity.

## 5 Conclusion

Acupuncture has shown positive effects on certain serum immune and inflammatory biomarkers and FEV1 in pediatric asthma. It has not shown beneficial effects on FEV1/FVC. However, due to the low quality of evidence, further high-quality clinical trials are needed to validate these findings.

## Data Availability

The original contributions presented in the study are included in the article/Supplementary material, further inquiries can be directed to the corresponding authors.
