# Supplementary material for: Effectiveness and safety of acupuncture and related therapies for pediatric asthma: a systematic review and meta-analysis
Source: Front Med (Lausanne). 2025 Jul 16;12:1626830. doi: 10.3389/fmed.2025.1626830 (PMC12307200; doi:10.3389/fmed.2025.1626830)
Supplement: Supplementary file 1 [file Data_Sheet_1.docx]

| Effectiveness and Safety of Acupuncture and Related Therapies for Pediatric Asthma: A Systematic Review and Meta-Analysis  **Supplementary Material: Exclude study** | | |
| --- | --- | --- |
| Serial Number | Title | Reasons |
|  | Immunomodulatory effects of acupuncture in the treatment of allergic asthma | 1 |
|  | Combination of laser acupuncture and probiotics in children with asthma | 2 |
|  | PRIST法测定过敏性哮喘儿童电针治疗前后血清 IgE水平的消长 | 4 |
|  | Immunomodulatory effects of acupuncture in the treatment of allergic asthma: a randomized controlled study | 1 |
|  | Efficacy of acupuncture for chronic asthma: study protocol for a randomized controlled trial | 4 |
|  | 刺络与舒利迭联合应用控制小儿哮喘迁延期肺脾气虚证的临床观察 | 2 |
|  | 穴位埋线在治疗支气管哮喘中对免疫力的影响研究 | 1 |
|  | 揿针治疗儿童肺脾气虚型哮喘(缓解期)疗效观察in Chinese | 3 |
|  | Acupuncture and bronchial asthma:: A long-term randomized study of the effects of real versus sham acupuncture compared to controls in patients with bronchial asthma | 1 |
|  | [Effects of acupuncture for dispersing fei, invigorating pi and reinforcing shen on heart rate variability and pulmonary function in bronchial asthma patients] | 1 |
|  | [Effects of acupuncture on clinical symptoms and pulmonary function in the patient of bronchial asthma] | 1 |
|  | Integrative medicine and asthma | 4 |
|  | Laser acupuncture and probiotics in school age children with asthma: a randomized placebo-controlled pilot study of therapy guided by principles of Traditional Chinese Medicine. (vol 18, pg 160, 2007) | 2 |
|  | [Effects of acupuncture on the pulmonary function and heart rate variability in different state of bronchial asthma | 1 |
|  | Complementary and Alternative Medicine Use and Adherence With Pediatric Asthma Treatment | 4 |
|  | [Controlled observation of non-blister acupoint sticking and electroacupuncture for bronchial asthma] | 3 |
|  | Observation on Chinese Herbs Combined with Acupuncture in Treating the Remission Stag of Children Asthma | 1 |
|  | Clinical observation on child asthma with heat syndrome treated by needle therapy | 3 |
|  | A randomized, controlled, crossover study in patients with mild and moderate asthma undergoing treatment with traditional Chinese acupuncture | 1 |
|  | Efficacy of acupuncture for chronic asthma: study protocol for a randomized controlled trial | 4 |
|  | Assessment of Acupoint Therapy of Traditional Chinese Medicine on Cough Variant Asthma: A Meta-analysis (Retracted Article) | 4 |
|  | innovative approach to laser acupuncture therapy of acute obstruction in asthmatic children | 1 |
|  | 激光穴位针灸联合普米克令舒治疗儿童支气管哮喘的疗效观察及对炎性因子和细胞免疫的影响 | 1 |
|  | Observation on the therapeutic effect of acupuncture combined with vitamin AD in the treatment of children with asthma and recurrent respiratory tract infection | 1 |
|  | [Acupuncture - has it a demonstrable bronchospasmolytic effect in bronchial asthma (author's transl)] | 4 |
|  | Effect of acupuncture on bronchial asthma | 4 |
|  | Acupuncture in bronchial asthma: bodyplethysmographic measurements of acute bronchospasmolytic effects | 4 |
|  | Comparison of real and simulated acupuncture and isoproterenol in methacholine-induced asthma | 4 |
|  | A controlled trial of real and simulated acupuncture in the management of chronic asthma | 1 |
|  | Efficacy of laser-acupuncture in the prevention of exercise-induced asthma | 1 |
|  | Clinical observation on 25 cases of hormone dependent bronchial asthma treated by acupuncture | 1 |
|  | 针灸联合尘螨变应原特异性免疫治疗儿童哮喘合并鼻炎的临床观察 | 1 |
|  | Acupuncture and bronchial asthma: a long-term randomized study of the effects of real versus sham acupuncture compared to controls in patients with bronchial asthma | 1 |
|  | [Observation on effect of integrative Chinese and Western medicine in treating acute attack of bronchial asthma] | 1 |
|  | [Clinical efficacy of target low-intensity laser radiation on the adrenal projection region in patients with bronchial asthma] | 2 |
|  | [Outpatient use of laser therapy in bronchial asthma] | 2 |
|  | [Low-intensity laser radiation in therapy of bronchial asthma] | 2 |
|  | [Effects of acupuncture on clinical symptoms and pulmonary function in the patient of bronchial asthma] | 1 |
|  | [Effects of acupuncture for dispersing fei, invigorating pi and reinforcing shen on heart rate variability and pulmonary function in bronchial asthma patients] | 1 |
|  | [Intra-auricular laser therapy of children suffering from bronchial asthma] | 6 |
|  | Laser acupuncture and probiotics in school age children with asthma: a randomized, placebo-controlled pilot study of therapy guided by principles of Traditional Chinese Medicine | 2 |
|  | [Effects of acupuncture on the pulmonary function and heart rate variability in different state of bronchial asthma] | 1 |
|  | Observation of the effect of acupuncture combined with medication on children with allergic rhinitis and asthma syndrome and its influence on FeNO, FEV1% and PEF | 1 |
|  | Efficacy observation of needle embedding at auricular points plus medications for combined allergic rhinitis and asthma syndrome in children and its effects on pulmonary function, serum IgE and EOS, and FeNO | 1 |
|  | [Effect of integrated traditional Chinese and Western medicine on Th1/Th2 cytokines level in children with asthma] | 2 |
|  | [Electroacupuncture at back-shu points as main combined with solar-term moxibustion for 150 cases of bronchial asthma] | 1 |
|  | [Observation on effect of acupuncture at Yuji (LU 10) on the pulmonary function of patients with bronchial asthma and immediate efficacy of relieving asthma] | 1 |
|  | Complementary and alternative medicine in children: an analysis of the recent literature | 4 |
|  | Laser acupuncture in treatment of childhood bronchial asthma | 4 |
|  | Surveys of complementary and alternative medicine usage: a scoping study of the paediatric literature | 4 |
|  | [Controlled observation of non-blister acupoint sticking and electroacupuncture for bronchial asthma] | 3 |
|  | [Impacts on asthma at persistent stage and immune function in the patients treated with acupuncture for warming yang and benefiting qi] | 1 |
|  | Efficacy of acupuncture for chronic asthma: study protocol for a randomized controlled trial | 4 |
|  | Acupuncture in children and adolescents with bronchial asthma A randomized controlled trial | 7 |
|  | 穴位埋线治疗小儿咳嗽变异性哮喘的疗效观察 | 7 |
|  | Acupuncture in Asthmatic Children: A Prospective, Randomized, Controlled Clinical Trial of Efficacy | 2 |
|  | 温阳扶正方针刺联合艾灸治疗儿童支气管哮喘慢性持续期的临床疗效观察 | 2 |
|  | Effect of acupuncture and its influence on cerebral activity in patients with persistent asthma: study protocol for a randomized controlled clinical trial | 4 |
|  | Pengobatan asma bronkial pada anak dengan akupunktur (the treatment of brondial asthma by acupuncture) | 5 |
|  | Laser acupuncture in children and adolescents with exercise induced asthma | 4 |
|  | 中药内服联合穴位针灸治疗小儿哮喘缓解期的效果研究 | 2 |
|  | 穴位埋线联合麻杏石甘茶治疗小儿咳嗽变异性哮喘的临床研究 | 2 |
|  | 火针对支气管哮喘IgE、IFN-γ和IL-4影响的临床研究 | 1 |
|  | 飞针疗法治疗小儿热证哮喘急性发作的临床研究 | 3 |
|  | 刺络联合吸入疗法防治小儿哮喘肺脾两虚型临床观察 | 2 |
|  | 刺络联合舒利迭吸入疗法防治小儿哮喘的疗效观察 | 2 |
|  | 针药并用治疗儿童过敏性鼻炎-哮喘综合征的疗效观察及对FeNO、FEV1%、PEF的影响 | 1 |
|  | 蜂针治疗儿童哮喘的临床疗效及对免疫功能的影响 | 2 |
|  | Effects of Acupuncture-like Transcutaneous Electrical Nerve Stimulation  on Children with Asthma | 2 |
|  | 针刺治疗对支气管哮喘急性发作期患者肠道菌群影响（ChiCTR2000040152） | 1 |
|  | 循经取穴针刺干预慢性持续期哮喘的中枢机制研究ChiCTR1900027478 | 1 |
|  | 循经取穴针刺干预慢性持续期哮喘的临床研究ChiCTR1900027284 | 1 |
|  | 针刺治疗哮喘的临床研究ChiCTR-OOC-17011086 | 1 |
|  | 哮喘患者痛觉异常对电针疗效影响的动态脑效应机制研究ChiCTR2300077503 | 1 |
|  | Research on Traditional Chinese Medicine (TCM) Clinical Treatment of the Acute Episode of Bronchial Asthma. NCT01293734 | 1 |
|  | Clinical Assessment of Acupuncture for the Treatment of Chronic Asthma. NCT01931696 | 1 |
|  | Acupuncture for Rhinitis Complicated With Asthma. NCT02033252 | 1 |
|  | Effectiveness of Acupuncture for Asthma. NCT00917215 | 1 |
|  | Characterization of Placebo Responses in Stable Asthma, NCT01143688 | 1 |
|  | Effect of Acu-Transcutaneous Electrical Nerve Stimulation (TENS) on Post-Exercise Expiratory Flow Rate in Subjects With Asthma. NCT00824642 | 1 |

The following are five reasons for literature exclusion:

1. Improper participants (n = 39)

2. Improper intervention (n = 16)

3. Improper control (n = 5)

4. Improper study design (n = 16)

5. Improper outcomes (n = 1)

6. Not full text (n = 1)

7. Repeated publication of literature (n = 2)
