# Supplementary material for: Effectiveness and safety of acupuncture and related therapies for pediatric asthma: a systematic review and meta-analysis
Source: Front Med (Lausanne). 2025 Jul 16;12:1626830. doi: 10.3389/fmed.2025.1626830 (PMC12307200; doi:10.3389/fmed.2025.1626830)
Supplement: Supplementary file 2 [file Data_Sheet_2.docx]

Effectiveness and Safety of Acupuncture and Related Therapies for Pediatric Asthma: A Systematic Review and Meta-Analysis

**Supplementary Material: Search strategy**

| **Database** | **Search strategy** |
| --- | --- |
| Pubmed | 1. "Asthma"[Mesh] 2. "Asthmas"[Title/Abstract] OR "Bronchial Asthma"[Title/Abstract] OR "Asthma, Bronchial"[Title/Abstract] 3. #1 OR #2 4. "child"[Mesh] OR "infant"[Mesh] OR "adolescent"[Mesh] 5. "child"[Title/Abstract] OR "pediatric"[Title/Abstract] OR "newborn"[Title/Abstract] OR "neonate"[Title/Abstract] OR "infant"[Title/Abstract] OR "preschool"[Title/Abstract] OR "adolescent"[Title/Abstract] OR "teenager"[Title/Abstract] OR "youth"[Title/Abstract] OR "juvenile"[Title/Abstract] OR "child*"[Title/Abstract] OR "pediatric*"[Title/Abstract] OR "newborn*"[Title/Abstract] OR "neonate*"[Title/Abstract] OR "infant*"[Title/Abstract] OR "preschool*"[Title/Abstract] OR "adolescent*"[Title/Abstract] OR "teenager*"[Title/Abstract] OR "youth*"[Title/Abstract] OR "juvenile*"[Title/Abstract] 6. #4 OR #5 7. "Medicine, Chinese Traditional"[Mesh] OR "Complementary Therapies"[Mesh] OR "Acupuncture"[Mesh] OR "Acupuncture Therapy"[MesH] OR "acupuncture, ear"[MesH] OR "electroacupuncture"[MesH] 8. "acupuncture*"[Title/Abstract] OR "electro stimulation"[Title/Abstract] OR "electric stimulation"[Title/Abstract] OR "auricular*"[Title/Abstract] OR "laser therapy"[Title/Abstract] OR "transcutaneous"[Title/Abstract] OR "acupoint application"[Title/Abstract] OR "acupoint stimulation"[Title/Abstract] OR "auricular point sticking"[Title/Abstract] OR "acupressure*"[Title/Abstract] OR "acupuncture"[Title/Abstract] OR "Acupuncture Therapy"[Title/Abstract] OR "Auricular points"[Title/Abstract] OR "Ear Acupuncture"[Title/Abstract] OR "electroacupuncture"[Title/Abstract] OR "needle"[Title/Abstract] OR "needling"[Title/Abstract] OR "prick"[Title/Abstract] OR "pricking"[Title/Abstract] OR "quick puncture"[Title/Abstract] 9. #7 OR #8 10. ((clinical[Title/Abstract] AND trial[Title/Abstract]) OR clinical trials as topic[MeSH Terms] OR clinical trial[Publication Type] OR random*[Title/Abstract] OR random allocation[MeSH Terms]) 11. #3 AND #6 AND #9 AND #10 |
| Web of Science | 1. TS = ("Asthma" OR "Bronchial Asthma" OR "Asthmas") 2. TS = ("child" OR "pediatric" OR "newborn" OR "neonate" OR "infant" OR "preschool" OR "adolescent" OR "teenager" OR "youth" OR "juvenile" OR "child" OR "infant" OR "adolescent" OR "child*" OR "pediatric*" OR "newborn*" OR "neonate*" OR "infant*" OR "preschool*" OR "adolescent*" OR "teenager*" OR "youth*" OR "juvenile*") 3. TS = ("Medicine, Chinese Traditional" OR "Complementary Therapies" OR "acupuncture*" OR "electro stimulation" OR "electric stimulation" OR "auricular*" OR "laser therapy" OR "transcutaneous" OR "acupoint application" OR "acupoint stimulation" OR "auricular point sticking" OR "acupressure*" OR "Acupuncture" OR "Acupuncture Therapy" OR "acupuncture, ear" OR "electroacupuncture" OR "acupuncture" OR "Acupuncture Therapy" OR "Auricular points" OR "Ear Acupuncture" OR "electroacupuncture" OR "needle" OR "needling" OR "prick" OR "pricking" OR "quick puncture") 4. TS = (("clinical" AND "trial") OR "random allocation" OR "random*") 5. #1 AND #2 AND #3 AND #4 |
| Cochrane Library | - 1. (asthma OR Bronchial Asthma OR Asthmas):ti,ab,kw   2. (child OR pediatric OR newborn OR neonate OR infant OR preschool OR adolescent OR teenager OR youth OR juvenile OR child OR infant OR adolescent OR child* OR pediatric* OR newborn* OR neonate* OR infant* OR preschool* OR adolescent* OR teenager* OR youth* OR juvenile*):ti,ab,kw   3. (Medicine, Chinese Traditional OR Complementary Therapies OR acupuncture* OR electro stimulation OR electric stimulation OR auricular* OR laser therapy OR transcutaneous OR acupoint application OR acupoint stimulation OR auricular point sticking OR acupressure* OR Acupuncture OR Acupuncture Therapy OR acupuncture, ear OR electroacupuncture OR acupuncture OR Acupuncture Therapy OR Auricular points OR Ear Acupuncture OR electroacupuncture OR needle OR needling OR prick OR pricking OR quick puncture*):ti,ab,kw   4. ((trial AND clinical) OR random allocation OR random):ti,ab,kw   5. #1 AND #2 AND #3 AND #4 |
| Embase | 1. 'asthma':ab,ti OR 'Bronchial Asthma':ab,ti OR 'Asthmas':ab,ti 2. 'child':ab,ti OR 'pediatric':ab,ti OR 'newborn':ab,ti OR 'neonate':ab,ti OR 'infant':ab,ti OR 'preschool':ab,ti OR 'adolescent':ab,ti OR 'teenager':ab,ti OR 'youth':ab,ti OR 'juvenile':ab,ti OR 'child':ab,ti OR 'infant':ab,ti OR 'adolescent':ab,ti OR 'child*':ab,ti 3. 'Medicine, Chinese Traditional':ab,ti OR 'Complementary Therapies':ab,ti OR 'acupuncture*':ab,ti OR 'electro stimulation':ab,ti OR 'electric stimulation':ab,ti OR 'auricular*':ab,ti OR 'laser therapy':ab,ti OR 'transcutaneous':ab,ti OR 'acupoint application':ab,ti OR 'acupoint stimulation':ab,ti OR 'auricular point sticking':ab,ti OR 'acupressure*':ab,ti OR 'Acupuncture':ab,ti OR 'Acupuncture Therapy':ab,ti OR 'acupuncture, ear':ab,ti OR 'electroacupuncture':ab,ti OR 'acupuncture':ab,ti OR 'Acupuncture Therapy':ab,ti OR 'Auricular points':ab,ti OR 'Ear Acupuncture':ab,ti OR 'electroacupuncture':ab,ti OR 'needle':ab,ti OR 'needling':ab,ti OR 'prick':ab,ti OR 'pricking':ab,ti OR 'quick puncture' 4. ('trial':ab,ti AND 'clinical':ab,ti)' OR 'random allocation':ab,ti OR 'random*':ab,ti 5. #1 AND #2 AND #3 AND #4 |
| China National Knowledge Infrastructure （CNKI） | 1. SU=哮喘 2. SU= (儿童 + 小儿 + 患儿 + 新生儿 + 婴儿 + 幼儿 + 婴幼儿 + 学龄前 + 少年 + 青少年 + 儿) 3. SU= (针灸 + 针刺 + 电针 + 温针灸 + 火针 + 穴位 + 耳针 + 毫针 + 针法 + 刺法 + 头针 + 刺络放血 + 针刀 + 腹针 + 浮针 + 梅花针 + 经皮穴位电刺激) 4. (SU='随机' OR FT='随机' OR FT='试验' OR FT='观察' OR FT='疗效评价') 5. #1 AND #2 AND #3 AND #4 |
| Database for Chinese Technical Periodicals (VIP) | 1. M=哮喘 2. M=（儿童 OR 小儿 OR 患儿 OR 新生儿 OR 婴儿 OR 幼儿 OR 婴幼儿 OR 学龄前 OR 少年 OR 青少年 OR 儿） 3. M=（针灸 OR 针刺 OR 电针OR 火针 OR 穴位 OR 耳针 OR 毫针 OR 针法 OR 刺法 OR 头针 OR 刺络放血 OR 针刀 OR 腹针 OR 浮针 OR 梅花针 OR 经皮穴位电刺激） 4. M=（试验 OR 观察 OR 随机 OR 疗效评价） 5. #1 AND #2 AND #3 AND #4 |
| Wanfang Database | 1. 主题: 哮喘 2. 主题: （儿童 OR 小儿 OR 患儿 OR 新生儿 OR 婴儿 OR 幼儿 OR 婴幼儿 OR 学龄前 OR 少年 OR 青少年 OR 儿） 3. 主题:（针灸 OR 针刺 OR 电针OR 火针 OR 穴位 OR 耳针 OR 毫针 OR 针法 OR 刺法 OR 头针 OR 刺络放血 OR 针刀 OR 腹针 OR 浮针 OR 梅花针 OR 经皮穴位电刺激） 4. 主题:（试验 OR 观察 OR 随机 OR 疗效评价） 5. #1 AND #2 AND #3 AND #4 |
| Chinese Biomedical Literature Database | 1. 哮喘 2. （儿童 OR 小儿 OR 患儿 OR 新生儿 OR 婴儿 OR 幼儿 OR 婴幼儿 OR 学龄前 OR 少年 OR 青少年 OR 儿） 3. （针灸 OR 针刺 OR 电针OR 火针 OR 穴位 OR 耳针 OR 毫针 OR 针法 OR 刺法 OR 头针 OR 刺络放血 OR 针刀 OR 腹针 OR 浮针 OR 梅花针 OR 经皮穴位电刺激） 4. （试验 OR 观察 OR 随机 OR 疗效评价） 5. #1 AND #2 AND #3 AND #4 |
